# The Effect of Substrate Stiffness on Elastic Force Transmission in the Epithelial Monolayers over Short Timescales

**DOI:** 10.1007/s12195-023-00772-0

**Published:** 2023-07-13

**Authors:** Aapo Tervonen, Sanna Korpela, Soile Nymark, Jari Hyttinen, Teemu O. Ihalainen

**Affiliations:** 1 BioMediTech, Faculty of Medicine and Health Technology, Tampere University, Arvo Ylpön katu 34, 33520 Tampere, Finland; 2 Department of Biological and Environmental Science, Faculty of Mathematics and Science, University of Jyväskylä, Survontie 9 C, 40500 Jyväskylä, Finland

**Keywords:** Mechanobiology, Epithelium, ECM stiffness, Computational modeling, Cell micromanipulation

## Abstract

**Purpose:**

The importance of mechanical forces and microenvironment in guiding cellular behavior has been widely accepted. Together with the extracellular matrix (ECM), epithelial cells form a highly connected mechanical system subjected to various mechanical cues from their environment, such as ECM stiffness, and tensile and compressive forces. ECM stiffness has been linked to many pathologies, including tumor formation. However, our understanding of the effect of ECM stiffness and its heterogeneities on rapid force transduction in multicellular systems has not been fully addressed.

**Methods:**

We used experimental and computational methods. Epithelial cells were cultured on elastic hydrogels with fluorescent nanoparticles. Single cells were moved by a micromanipulator, and epithelium and substrate deformation were recorded. We developed a computational model to replicate our experiments and quantify the force distribution in the epithelium. Our model further enabled simulations with local stiffness gradients.

**Results:**

We found that substrate stiffness affects the force transduction and the cellular deformation following an external force. Also, our results indicate that the heterogeneities, e.g., gradients, in the stiffness can substantially influence the strain redistribution in the cell monolayers. Furthermore, we found that the cells’ apico-basal elasticity provides a level of mechanical isolation between the apical cell–cell junctions and the basal focal adhesions.

**Conclusions:**

Our simulation results show that increased ECM stiffness, e.g., due to a tumor, can mechanically isolate cells and modulate rapid mechanical signaling between cells over distances. Furthermore, the developed model has the potential to facilitate future studies on the interactions between epithelial monolayers and elastic substrates.

**Supplementary Information:**

The online version of this article (10.1007/s12195-023-00772-0) contains supplementary material, which is available to authorized users.

## Introduction

Our understanding of the importance of mechanical forces and microenvironment in cellular processes and signaling alongside biochemistry has drastically improved during the last decades [42, 67]. Cellular mechanics have a vital role, for example, in embryogenesis, stem cell differentiation, tissue homeostasis, and cell migration [6, 15, 23, 62, 70].

Mechanically cells can be considered as an active soft material which responds, generates, and transmits forces. They respond to mechanical forces mainly elastically over a short timescale of seconds by deforming, and viscously over sustained stress by dissipating the stress via several processes (e.g., neighbor exchange and oriented cell division) at the timescales of minutes to tens of minutes [9, 32, 56]. Cells use their actomyosin cytoskeleton to generate contractile forces that enable them to change their shape and move [7, 34]. Since the actomyosin cytoskeletons of neighboring epithelial cells are connected via adherens junctions, these contractile forces can be transmitted between cells over long distances [52, 57, 62, 66]. In addition, cells connect their cytoskeleton to the ECM mainly via focal adhesions, structures that enable the cells to sense the external forces and ECM stiffness [12, 68]. Mechanical forces can also be converted to biochemical signals by the cells in a process termed mechanotransduction [67] or transmitted directly to the nucleus where the can modulate gene expression [29, 35]. Timescales of these cellular force responses vary, some happening in less than a second (e.g., Rac1 activation), while others can take tens of minutes (e.g., structural changes in fibronectin) [44, 53].

Due to the variety of cellular responses, it is not surprising that external forces and physical properties of the cell environment have been shown to affect many cellular functions during development, homeostasis, and diseases. For example, ECM stiffness has been linked to cell differentiation [15] and the metastatic potential of tumors [13, 48]. It is well established that tumor stroma, the non-cancer cell containing structural component of the tumor, is often considerably stiffer than native tissue [8, 14]. This leads to stiffness gradients and interfaces between the tumor and the surrounding healthy tissue, which can influence cellular mechanosignaling, especially during cancer invasion [1, 31]. Furthermore, stiffness gradients can be present also within tumors, e.g., some ovarian cancers show stiffer core in comparison to periphery of the tumor [38]. Thus, the effects of stiffness, and stiffness gradients and interfaces on mechanosignaling and mechanotransduction during the tumor progression are currently actively studied themes. Here, computational modeling together with simplified multicellular models, e.g., epithelial monolayers, provides much needed simplified tools to understand these highly complex processes.

There is a plethora of computational methods that can describe the mechanical system formed by the epithelial monolayer. Vertex models are a relatively simplistic approach that reduces the mechanical properties of the cells to only a few parameters [18, 50]. Methods such as the subcellular element method [30, 41] and immersed-boundary method [46, 59] provide a more nuanced description of the cells and their mechanical properties. Deformable substrate has been included in some single cell migration models as well as in some 1D or 2D models of collective epithelial behavior [16, 25, 30, 33, 50]. However, to our knowledge, there currently is no model that combines the fine description of the subcellular structures and mechanics with a deformable substrate in the context of an epithelial monolayer.

This study aimed to describe how strain and forces spread in an elastic mechanical system formed by the epithelial monolayer on deformable substrates with varying stiffnesses over short timescales. The work was conducted experimentally using Madin–Darby canine kidney (MDCK) II cell model on elastic polyacrylamide (PAA) hydrogel substrates and computationally using a cell-based modeling framework we developed. To investigate the transmission of forces in this system, we—experimentally and in the computational model—mechanically manipulated a single cell in the epithelium, causing a local stretching in the epithelium and the substrate. Our computational model was further used to study the effect of local changes in the substrate stiffness on force transduction as well as the effect of substrate stiffness on subcellular changes in cell shapes. As a part of the computational approach, we developed a graphical user interface for the model to allow easier usage of the platform.

## Results

### Micromanipulation of Epithelial Monolayers on Substrates with Varying Stiffness

**Fig. 1:**
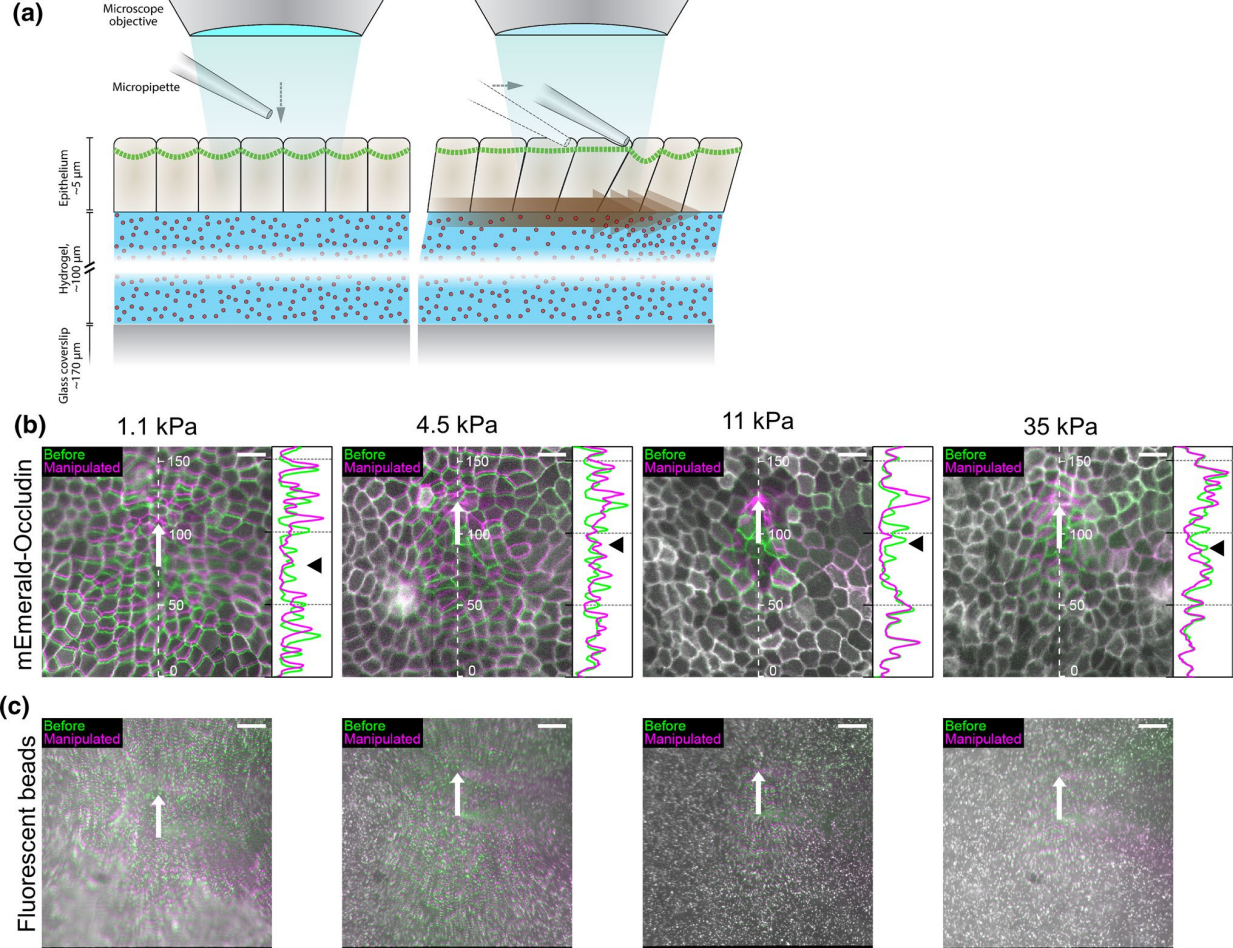
**a** The manipulation of a single cell with a piezo-driven micropipette while imaging. First, the pipette was lowered to the target cell in order to be manipulated. Next, the pipette was moved 30 $\upmu$m parallel to the epithelium surface and the deformation of the surrounding cells and the underlying substrate was observed. **b** Representative examples of mEmerald-Occludin-expressing MDCK II cell movement on polyacrylamide (PAA) hydrogel substrates with stiffnesses of 1.1, 4.5, 11, and 35 kPa following the movement of the micromanipulated pipette for 30 $\upmu$m in 1 s (pipette movement shown by the white arrow). The boundaries of cells were indicated using mEmerald-Occludin and shown in green before the micromanipulation and in magenta following the 30 $\upmu$m pipette movement. The cell displacement on the right side of the pipette (white arrow) is partly due to the pipette affecting part of the image. The line plots show the cell boundaries before (green) and following the micromanipulation (magenta) for each gel stiffness along the dashed lines to better show the magnitude of the cell movement along the pipette movement axis. The data was smoothed using 10 pixel moving average. The initial pipette position is indicated by the black arrow heads. **c** The movement of the fluorescent beads embedded in the PAA hydrogel substrates with the corresponding stiffness underlying the epithelia shown in **b**. The pipette movement of 30 $\upmu$m is shown by the white arrow and the bead locations before and following the micromanipulation in green and magenta, respectively. The pipette shadow was affecting the results on the right side of the white arrow. *Scale bars* 20 $\upmu$m

To experimentally study the effect of environmental stiffness on the transmission of elastic forces and deformation in epithelial tissue, we used an in vitro model of MDCK II cells expressing tight junction marker mEmerald-Occludin cultured on PAA hydrogel substrates with embedded fluorescent microbeads. We used collagen-I-coated PAA substrates with four stiffnesses (Young’s moduli): 1.1, 4.5, 11, and 35 kPa. The Poisson’s ratio of PAA is close to 0.5, i.e., nearly incompressible independent of the stiffness [18, 50, 54]. We manipulated a single cell with a micropipette attached to a piezo-driven micromanipulator as a mechanical stimulus (Fig. 1a). The pipette was first brought into contact with the cell with the micromanipulator, next the pipette was lowered a distance between a few hundred nanometers and 1 $\upmu$m, allowing the pipette to pull and move the manipulated cell horizontally from the cell–cell junctions. The micromanipulator was used to move the pipette 30 $\upmu$m parallel to the surface in 1 s (speed 30 $\upmu$m s$^{-1}$) while simultaneously capturing approximately 13 images of the movement of both the mEmerald-Occludin and the fluorescent beads. This allowed us to follow the rapid elastic response of the cells and the hydrogel, since the cells did not have time to adapt to the manipulation viscoelastically, e.g., by reorganizing their cytoskeleton or cell–ECM junctions.

The micromanipulation led to a large deformation of the epithelium and displacement of the cells and the PAA substrate. We visualized the movement by comparing the images of the epithelium and the substrate before and following the micromanipulation (Fig. 1b, c). It is clear from the cell boundary movements (Fig. 1b) that, as expected, the PAA stiffness profoundly affects the distance that the mechanical strain spreads around the manipulated cell. For example, the movement of the cell boundaries along the axis of the pipette movement (Fig. 1b side panels) shows that on the 1.1-kPa substrate, the cell boundary at the edge of the imaged field (approximately 80 $\upmu$m from the initial pipette position) moves 3.3 $\upmu$m. In contrast, on the 11- and 35-kPa substrates, the discernible cell boundary movement happened only at the distance of approximately 50 $\upmu$m along the axis of the movement (Fig. 1b).

The displacement of the substrate was naturally affected by its own stiffness. There was deformation in the whole imaged field for both the 1.1- and 4.5-kPa substrates (Fig. 1c) in the direction parallel to the pipette movement. However, perpendicular to the manipulation, the deformation was limited for the 4.5-kPa substrate compared to 1.1 kPa. The deformation was even smaller with the stiffer (11 and 35-kPa) substrates (Fig. 1c).

**Fig. 2:**
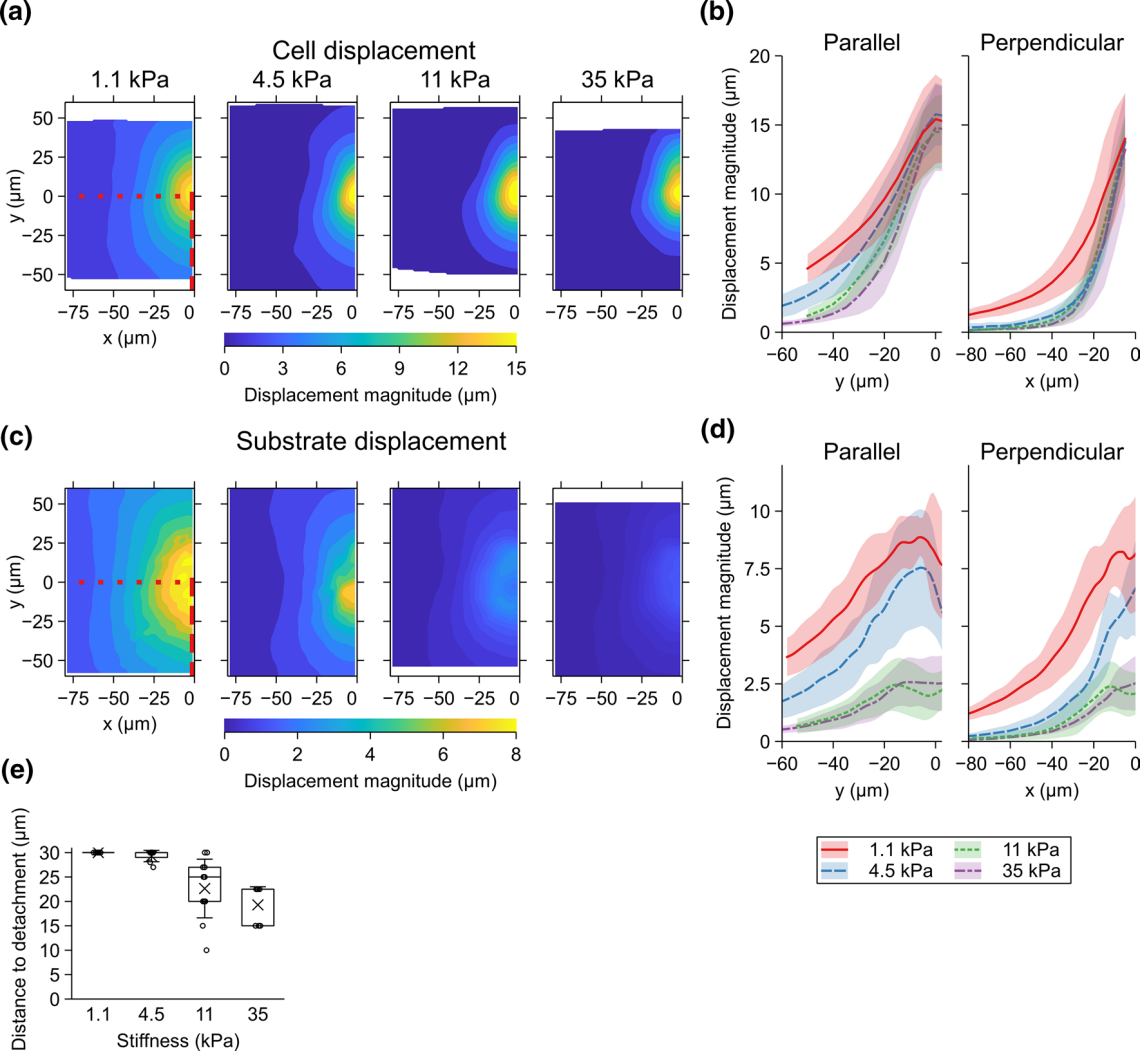
**a** Average displacement of the segmented MDCK II cell centers as function of location of the original cell positions in relation to the initial pipette position for the stiffnesses 1.1 (n = 11), 4.5 (n = 7), 11 (n = 11), and 35 kPa (n = 7). The field is limited to the left of micromanipulation axis since the movement was symmetric on either side of the axis. The area of the shown displacement field varies between the stiffnesses since the initial pipette position in relation to the imaging area varied between measurements. **b** The cell center displacement along the y-axis (red dashed line in **a**) parallel to the direction of the pipette movement (left) and along x-axis (red dotted line in **a**) perpendicular to the pipette movement direction (right) for each stiffness. **c** Average displacement of PAA hydrogel substrates based on the particle image velocimetry (PIV) analysis as function of location in relation to the initial pipette position for the stiffnesses 1.1 (n = 11), 4.5 (n = 7), 11 (n = 11), and 35 kPa (n = 7). The field is limited to the left of micromanipulation axis since the movement was symmetric on either side of the axis and the pipette causes artifacts in the PIV data on the right side of the pipette. The area of the shown displacement field varies between the stiffnesses since the pipette position in relation to the imaging area varied between measurements. Note that maximum displacement is different compared to the cells. **d** The PAA substrate displacement along the red dashed line in **c** parallel to the direction of the pipette movement (left) and along the red dotted line in **c** perpendicular to the pipette movement direction (right) for each stiffness. The shaded region represents the SD for each stiffness. **e** Distance of pipette movement before cells detached from the substrate estimated from the live imaging data for the different stiffnesses 1.1 (n = 11), 4.5 (n = 7), 11 (n = 11), and 35 kPa (n = 7). The indicated cases with the distance to detachment of 30 $\upmu$m did not detach from the substrate during the experiment

To quantify the cell displacements, we segmented the cells from the images before and following the micromanipulation to obtain the cell outlines. Using the outlines, we defined a geometrical cell center, which we then used to measure the displacement of the individual cell during the manipulation. This created a spatial map of the cell center movements relative to the initial position of the pipette. We then interpolated this movement data over the whole imaging area to obtain a continuous distribution that was then averaged over the measurements for each substrate stiffness (Fig. 2a, b). In order to do the same for the substrate data, we used particle image velocimetry (PIV) analysis to find the displacement of the microbeads during the manipulation. Like the cell data, this was averaged and plotted in relation to the initial pipette position (Fig. 2c, d).

Interestingly, the 30-$\upmu$m pipette movement translated to a manipulated cell center movement of a similar range independent of the substrate stiffness with values of 15.4 ± 3.2, 15.8 ± 2.3, 14.5 ± 2.6, and 14.8 ± 3.1 $\upmu$m (mean ± SD), from the softest to the stiffest substrate. This difference between the pipette and cell movements can be explained by the deformation and stretching of the manipulated cell. In addition, the substantial deformation of the cells being pushed made accurate quantification of the displacement difficult. Therefore, we mainly concentrate our analysis on the area where the pipette pulled and stretched the cells and present the results mainly as a function of distance along the negative y-axis relative to the manipulated cell. For example, parallel to the pipette movement (Fig. 2b, along the red, dashed line in 2a), the cell centers move 5 $\upmu$m or more within a distance of 47, 34, 26, and 20 $\upmu$m from the initial pipette position respectively for 1.1-, 4.5-, 11-, and 35-kPa substrates. Conversely, the displacement perpendicular to the pipette movement extended further for the softest substrate compared to the three stiffer ones, for which it was approximately similar (Fig. 2b).

The amount of substrate displacement was considerably smaller than that of the cells (Fig. 2d). The maximum displacements, located near the manipulated cell, were, from the softest to the stiffest, 8.9 ± 0.8, 7.6 ± 2.5, 2.7 ± 1.0, and 2.6 ± 1.1 $\upmu$m. Therefore, the relative magnitude of the maximum substrate displacement compared to that of the cells corresponding to the stiffnesses from 1.1 to 35 kPa were 0.58, 0.48, 0.19, and 0.17. This difference in the maximum displacements is partly explained by the fact that the cell–cell junctions and the substrate-binding focal adhesions are separated by the cell height, indicating vertical cell deformation. In addition, the obtained substrate movement close to the manipulated cell were affected in the PIV analysis by the shadow of the pipette and a slight out-of-focus indentation caused by the pipette pushing the cells.

Another factor that explains the difference in the maximum displacement between the cells and the substrate was that the cells around the pipette detaching from the substrate during the 30-$\upmu$m micromanipulation. This the in 29%, 82%, and 100% of the measurements with the 4.5-, 11-, and 35-kPa substrates, respectively (Fig. 2e and Supplementary Video S1). No detachment was observed with the 1.1-kPa substrate. This can be explained by the reduced substrate movement with the higher stiffness, which led to more apico-basal strain, and thus stress, in the cells themselves. In addition, curiously, the variance in the detachment distance for the 11-kPa substrate was larger than in the others. No detachment of cells from each other was observed in the measurements.

### Computational Modeling of Force Transmission in the Epithelium

**Fig. 3:**
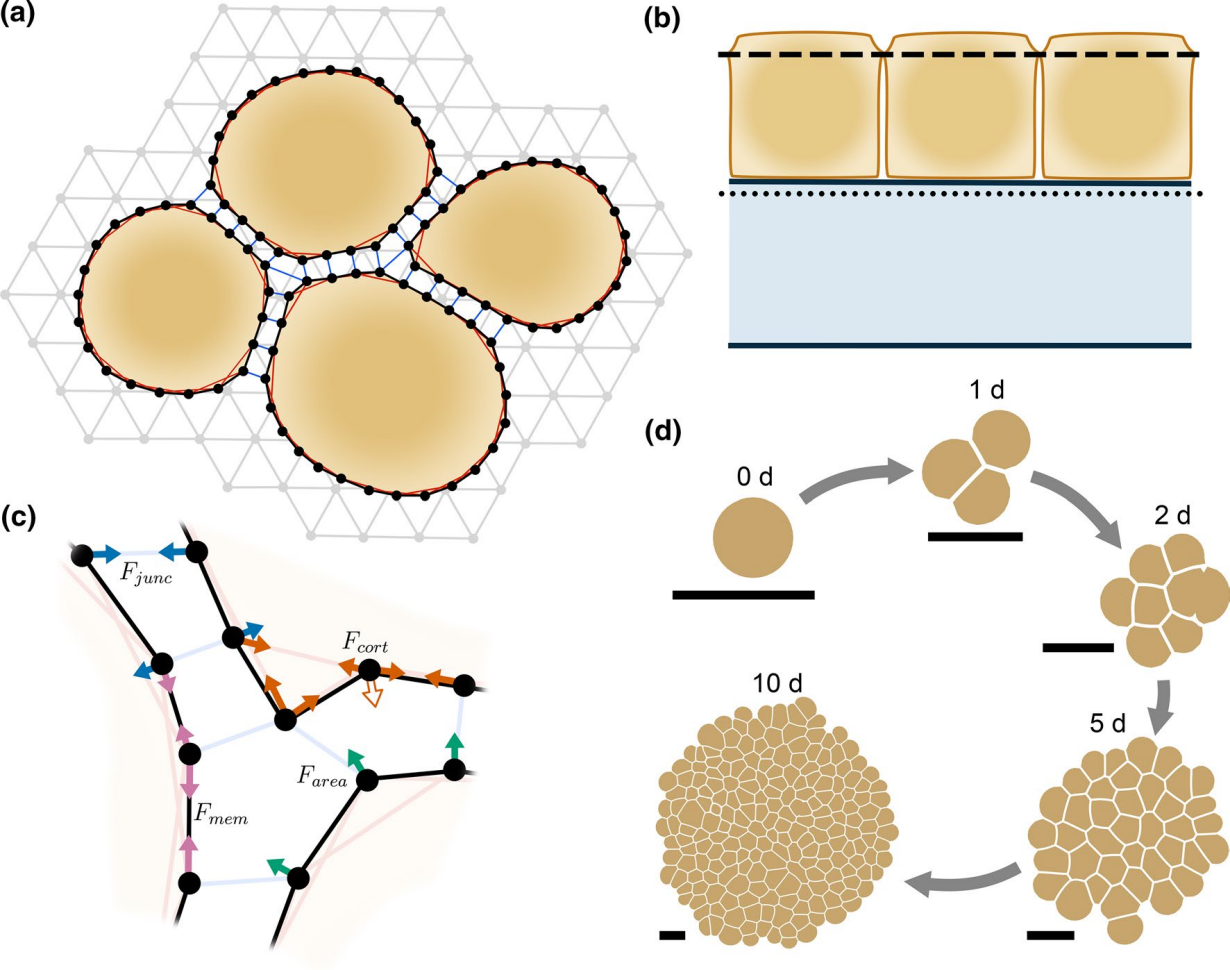
**a** Basic structure of the model. The cells were described by closed polygons and the cell structures and processes were included as forces affecting the polygon vertices. The cell–cell junctions and cortical actomyosin are depicted. The substrate was described by a triangular grid of points whose internal mechanics were defined by the forces between the grid points. The cell vertices were connected to the substrate via focal adhesion connections. **b** A side view of the model showing the level of the cell model with the dashed line at the height of the cell–cell junctions and the substrate model with the dotted line. **c** Example of forces that determine the cell vertex movements: cell–cell junction forces ($F_{junc}$), cortical forces ($F_{cort}$), membrane forces ($F_{mem}$), and intracellular pressure or area force ($F_{area}$). An additional cortical force component is added to the concave vertices, since the cortical link between its neighboring vertices runs behind it pushing it outwards (unfilled arrow). Note that the forces are calculated for every vertex but for simplicity, all forces are not shown for all vertices here. **d** Time series showing the growth of epithelial cell cluster from a single cell over a period of 10 days. *Scale bar* $\sim$ 20 $\upmu$m

In order to better understand the force transmission in the epithelial monolayers over short timescales, we developed a cell-based computational model to describe this mechanical system. The cells were constructed from closed 2D polygons, in which polygon vertices represented the cell–cell junctions (Fig. 3a, b). The model was evolved by calculating, largely Hookean, forces between the vertices (Fig. 3c) similar to the model by [58]. We described the deformable substrate under the cells as a triangular grid of points to represent its top surface (Fig. 3b) and whose movement was solved similar to that of the cell vertices. For a detailed explanation of the model, the fitting, and the simulations, see the description in the Supplementary Text. In addition, we created a graphical user interface for the modeling platform. We used our in vitro results and data from the literature to fit the model parameters. The computational model was first used to grow virtual epithelia (Fig. 3d), followed by the simulation of single-cell mechanical manipulation. During the simulated manipulation and similarly to the in vitro experiments, we assumed minimal remodeling of the mechanical properties of the cells within the experimental timescale to describe the system as purely elastic.

**Fig. 4:**
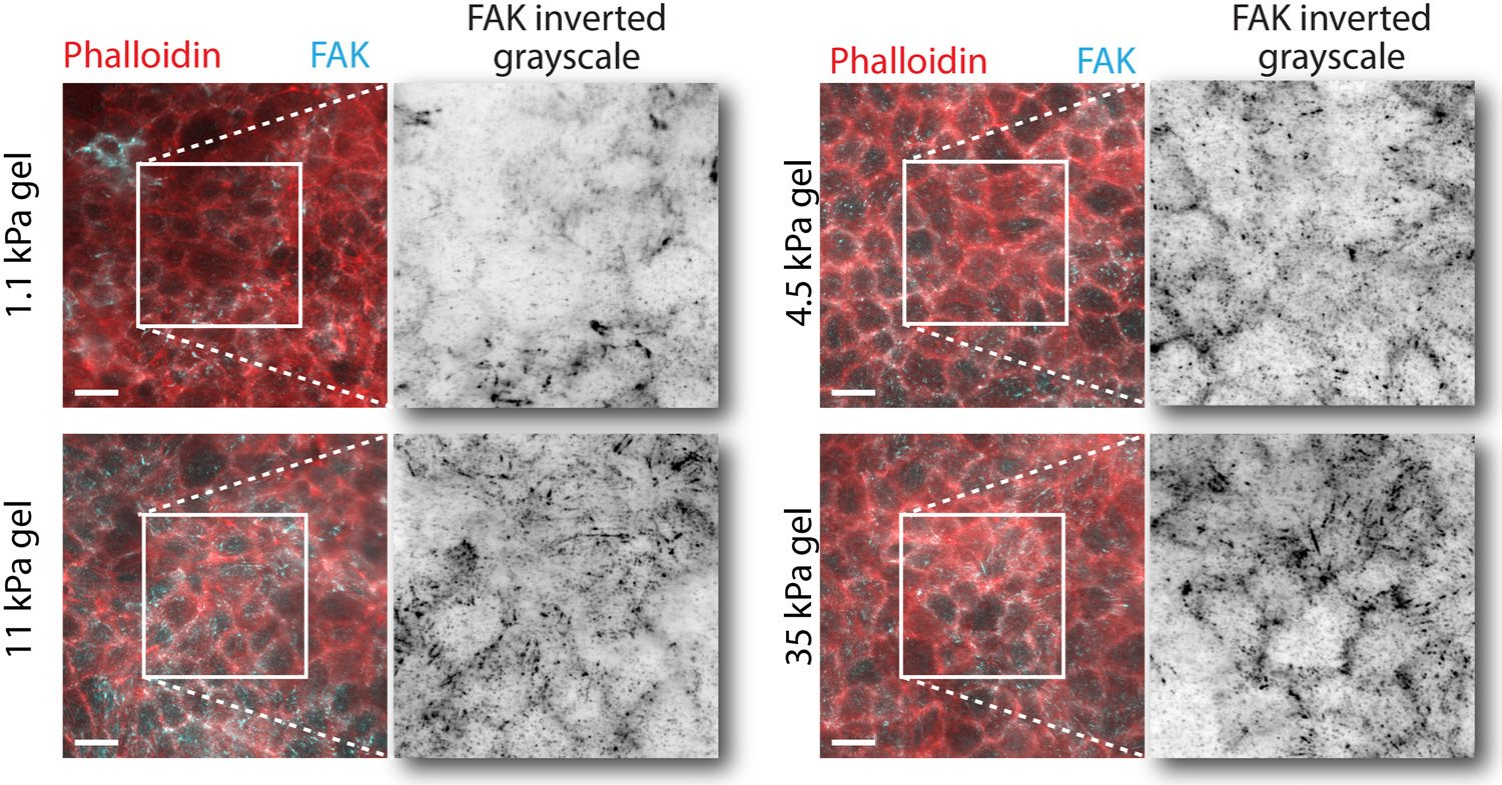
Cell boundaries (indicated by Phalloidin showing actin fibers) and focal adhesion kinases (FAKs) of epithelia grown on hydrogel substrate with stiffnesses 1.1, 4.5, 11, and 35 kPa. The FAK is shown in inverted grayscale for a zoomed in section. *Scale bars* 15 $\upmu$m

**Fig. 5:**
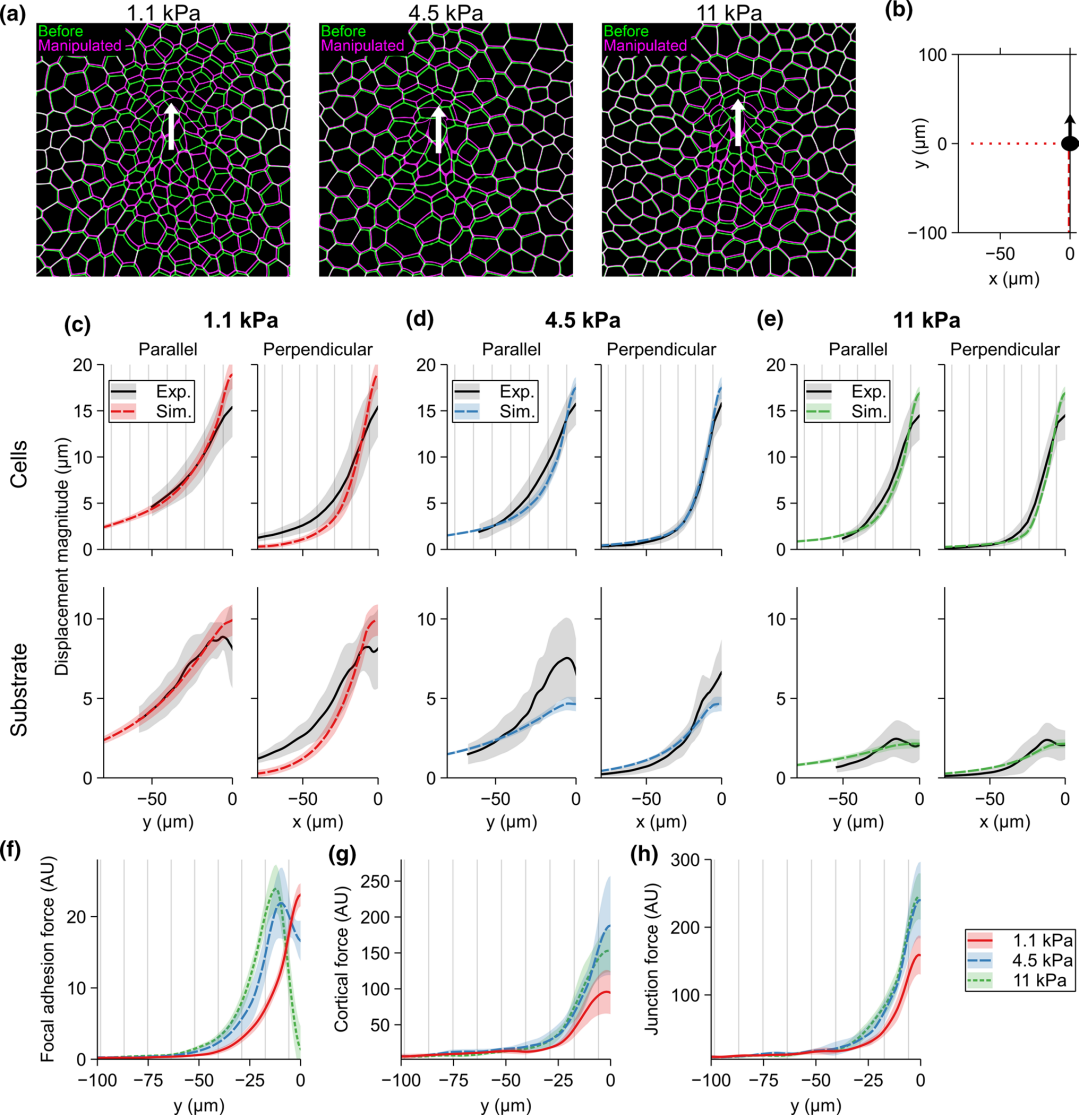
**a** Representative figures showing the cell displacement in the simulations during the micromanipulation for the 1.1-, 4.5-, and 11-kPa substrates highlighting the cell shapes before (green) and following the micromanipulation (magenta). The white arrow indicates the micromanipulator movement. The scale bars are 20 $\upmu$m. **b** Description of the axis the results were plotted on in **c**–**h**. Comparison between the experimental cell and substrate displacement with the fitted computational model for **c** 1.1, **d** 4.5, and **e** 11-kPa substrates. The top row for each stiffness shows the fit for cell displacement in parallel (left, dashed line in **b**) and perpendicular (right, dotted line in **b**) directions and the bottom row shows the same for the substrate displacements. **f** The focal adhesion, **g** the cortical, and **h** the junction forces parallel the pipette movement (dashed line in **a**) for the cells on 1.1-, 4.5-, and 11-kPa substrates in arbitrary units (AU). The force magnitudes are comparable between each other. The shaded region represents the SD. For each set of simulation parameters, n = 5

We fitted the model parameters by comparing the cell center and substrate displacements between the in vitro experiments and the computational model. This was done for stiffnesses 1.1, 4.5, and 11 kPa with all having the Poisson’s ratio of 0.5. We assumed that the substrate does not influence the elastic properties of the cells, and we used the same cellular parameter values for all substrate stiffnesses in the fitting process. This assumption is supported by the study by [47], where they measured cortical stiffness using atomic force microscopy and found it to be independent of the substrate stiffness. However, since increased substrate stiffness has been shown to induce more stable and stronger focal adhesions [71, 72], the related parameter (focal adhesion strength) was made to depend on the stiffness. We also confirmed this stiffness dependence by staining focal adhesion kinases (FAKs) with MDCKs grown on substrate with the difference stiffnesses. FAKs were more abundant in epithelia on stiffer substrates (Fig. 4), indicating higher focal adhesion strength and agreement with our assumption. Trial simulations for the 35-kPa substrate showed that the substrate model was unable to replicate the micromanipulations with this high stiffness independent of the focal adhesion strength (Supplementary Fig. S1). In addition, since the three softer substrates provided a large, 10-fold range in stiffness, the 35-kPa stiffness was omitted from the simulations. Like with the experiments, we focused on the area of the epithelium under tension in the simulation data analysis (i.e., $y < 0$, where the pipette is in the origin) (Fig. 5a, b).

The values obtained for the focal adhesion strengths were 0.5, 0.8, and 1.0 g/s^2^/$\upmu$m for 1.1, 4.5, and 11-kPa substrates, respectively. Since we described the focal adhesions by springs, the units include the unit of the spring constant (g/s^2^). In addition, the strength depends on the length of the membrane ($\upmu$m) that each cell vertex represents. Furthermore, it is essential to note that since the cell polygons represent the apical surface of the cells, the focal adhesion springs include the cell elasticity in the apico-basal axis. However, the increase in the focal adhesion strength as a function of substrate stiffness still reflects the stronger binding of the cell to a stiff substrate.

We analyzed the computational cell displacements similar to the experimental results by calculating the average cell center movement distributions in relation to the initial pipette position. The substrate displacement, on the other hand, was defined directly from the substrate point movements and transformed to be in relation to the initial pipette position and averaged over multiple simulations. The fitted model captured extremely well the general behavior of the experimental micromanipulation (Fig. 5a and Supplementary Video S2), especially in the region under tension (left side plots of Fig. 5c–e). However, the cells near the pipette showed higher displacement in the model for all stiffnesses, most likely due to the difficulty of cell segmentation in the experimental data in this area. Also, since these areas were affected in the PIV analysis, the substrate displacements here differ between the experiments and the model. The model underestimated the cell and substrate displacement perpendicular to the pipette movement direction with the 1.1-kPa substrate. Interestingly, the variabilities in the displacement between the computationally manipulated epithelia were small even though we ran each simulation using a different virtual epithelium.

Cell deformations are the result of forces acting on them. These forces are transmitted from the pipette into the wider epithelium via cell–cell junctions and cytoskeletons as well as to the substrate via focal adhesions [66]. Therefore, we were interested in how the different forces (Supplementary Fig. S2) are distributed within the epithelium depending on the substrate stiffness. To map the forces as a function in relation to the manipulated cell, we calculated the average force for each cell and assigned them to the original cell center positions. Next, we interpolated the averaged force magnitudes between the cell centers to obtain continuous spatial distributions and then averaged over multiple simulations.

We concentrated on three different force components: the cell–substrate interaction forces (focal adhesion forces, Fig. 5f), the elastic forces of the cell cortex opposing cell deformation (cortical force, Fig. 5g), and the cell–cell forces (junction forces, Fig. 5h). The substrate stiffness had an apparent effect on the maximum magnitudes of each of the three forces. The average maximum focal adhesion forces near the initial pipette position were 19.7, 27.6, and 24.6 AU (arbitrary units) from the softest 1.1 to the stiffest 11-kPa substrate (Fig. 5f). The maximum value for the 11-kPa substrate was affected by the cell detachment near the pipette. The corresponding average maximum values for the cortical forces were 83.5, 127.7, and 152.1 AU (Fig. 5g) and for the junction forces 133.2, 208.2, and 272.5 AU (Fig. 5h). There were only minor differences in the junction forces between the two stiffest substrates beyond the distance of 25 $\upmu$m from the initial pipette position. On the other hand, the focal adhesion forces showed the importance of the stiffness over the whole range of the simulated distance. For example, the focal adhesion force of 1 AU was sensed by the cells at distances of 42, 52, and 57 $\upmu$m on substrates from the softest to the stiffest, respectively (Fig. 5f).

The results indicate that the stiffness of the substrate had an apparent effect on the spreading of epithelial deformation following an external tensile force. However, while the junction and the cortical force of the cells were higher near the manipulated cell on stiffer substrates, there were only minor differences in the forces at longer distances. On the other hand, the focal adhesion forces on the softest substrate remained lowest over the simulated distance.

### Propagation of Forces over Substrate Stiffness Gradients

Next, we investigated how stiffness gradients—describing, e.g., those between stiff tumorous tissue and healthy soft tissue—affect the transduction of elastic forces between the cells. We concentrated on sudden changes in stiffness. While it is possible to produce PAA hydrogels with stiffness gradients [64], they generally have a shallow slope. On the other hand, micropillars have been used to create local stiffness boundaries [63, 65]. However, in this method the range of possible substrate displacement is limited, and the discrete nature of the substrate affects the cell adhesions [49]. Our computational model enabled the generation of epithelial monolayers attached to continuous substrates with stiffness gradients with sharp slopes.

#### From a Soft to a Stiff Substrate

**Fig. 6:**
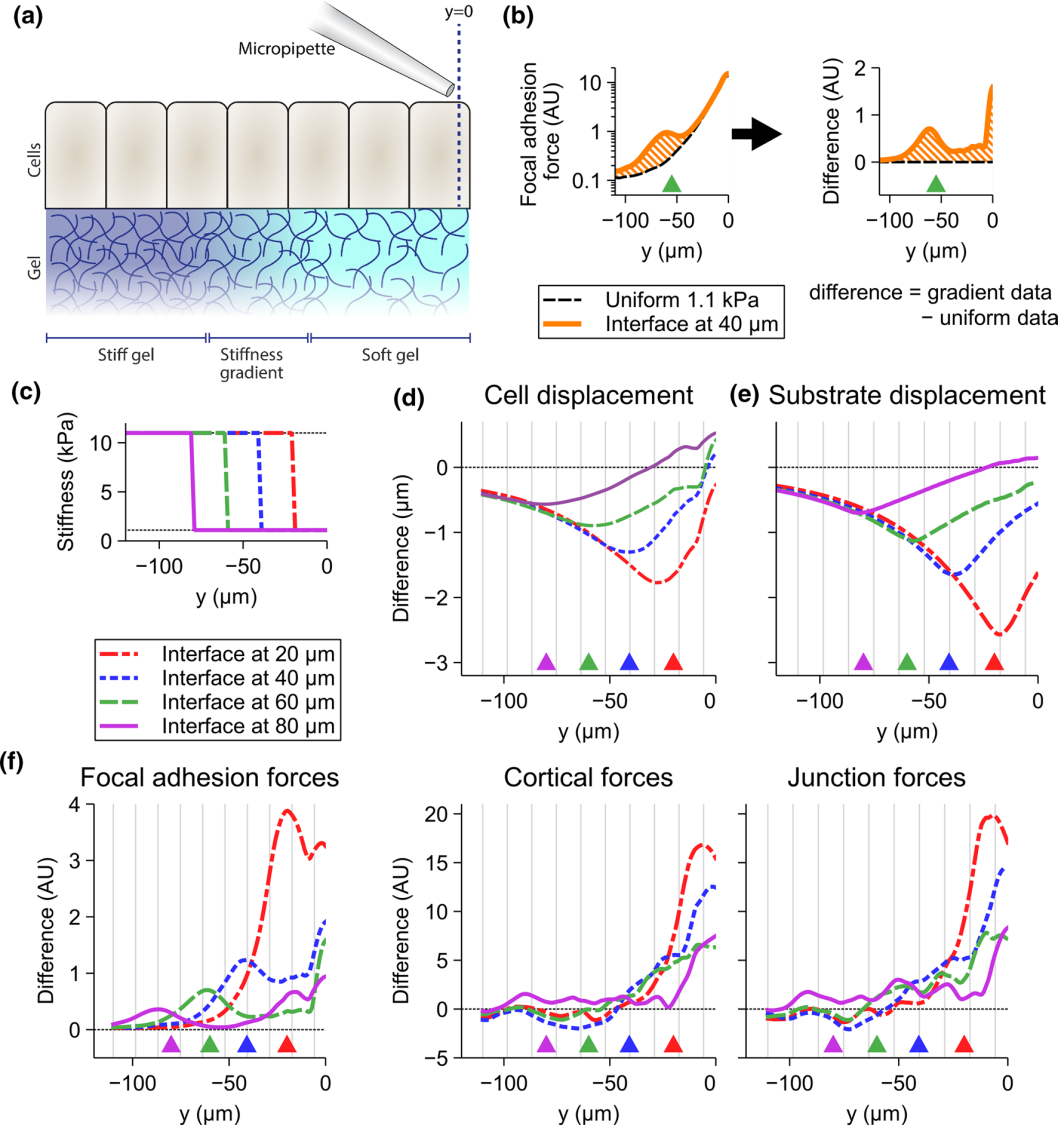
The spread of displacement and forces from soft substrate region to a stiff region. The result panels show the differences in vertical cell and substrate displacements and focal adhesion, cortical, and junction force transmissions caused by an interface gradient. The results are calculated towards the negative y-direction from the initial pipette position based on their average difference compared to the case where the substrate stiffness was the same as below the manipulated cell. **a** The manipulated cell is positioned at the initial pipette position [coordinates (0, 0)]. **b** Example of how the results are calculated for the focal adhesion forces for the stiffness interfaces at $y = -60 \upmu$m (green dashed line in right-most panel in **f**). The orange striped areas correspond to each other in the figure. **c** The stiffness interfaces for displacement and forces shown in **d**–**f**. The difference in **d** cell and **e** substrate displacement compared to the uniform 1.1-kPa displacement for stiffness interfaces at 20, 40, 60, and 80 $\upmu$m. **f** The differences in the average focal adhesion, cortical, and junction forces for the stiffness interfaces compared to the forces in the corresponding position with 1.1-kPa substrate. The magnitudes of the displacement and forces are shown in Supplementary Fig. S4. The vertical striping shows the positions of cell boundaries for average sized cells and the positions of the interfaces are shown with the arrowheads of corresponding colors at the bottom of each figure. For each case, n = 15. *AU* arbitrary unit

We first concentrated on how strain and forces spread from a soft substrate region to a stiff one as depicted in Fig 6a. To do this, we simulated the pipette micromanipulations with substrates containing one of the following three different types of stiffness gradients between 1.1 and 11 kPa: a stiffness interface (change in stiffness in 2 $\upmu$m, Fig. 6c), a sharp, or a shallow gradient (changes in stiffness in 10 $\upmu$m or 50 $\upmu$m, respectively, Supplementary Fig. S3). The pipette was moved only 20 $\upmu$m in these simulations to minimize cell detachment. We also simulated the 20-$\upmu$m micromanipulations with the uniform stiffnesses of 1.1 and 11 kPa for comparison. We analyzed the results by calculating how the cell and substrate displacements as well as the focal adhesion, cortical, and junction forces differed from the case with the uniform 1.1-kPa substrate. This was done by subtracting the resulting displacements and forces in the case with the rigidity gradient from the corresponding values obtained with the uniform rigidity. This is visualized by an example in Fig. 6b.

Compared to the 1.1-kPa uniform substrate, the rapid increase in stiffness at four different distances from the manipulated cell (20, 40, 60, or 80 $\upmu$m) led to a reduced cell displacement with the most prominent effect near the stiffness interface (Fig. 6d). While the reduction was more prominent when the interface was closer to the initial pipette position, it still occurred even when the interface was up to 80 $\upmu$m away. However, with the interface farther away, the difference in displacement remained below 1 $\upmu$m. The difference in the substrate displacement was slightly higher but otherwise similar to that of the cells (Fig. 6e). Thus, the soft-to-rigid substrate stiffness gradient reduced the cell and substrate displacement during simulated micromanipulation.

The decreased cell and substrate displacements were accompanied by increased forces (Fig. 6f). The cortical and junction forces were generally increased in 2–3 layers of cells (indicated by the vertical striping) from the manipulated cells towards the gradient interface. In some cases, e.g., with the interface being close to the manipulated cell, they remained increased beyond the interface. The focal adhesion forces were increased in the region between the manipulated cell and the interface, and there was a clear peak around the interface itself. Furthermore, they remained increased also well on the stiff substrate beyond the interface for 2–4 cell layers. While the difference peak in absolute terms was highest for the interface at 20 $\upmu$m from the initial pipette position, the relative increase was highest (over 5-fold) for the interface at 60 $\upmu$m. These peaks can be largely explained by the higher focal adhesion strength in the stiffer 11-kPa region. Thus, opposed to the displacements, the gradient increased the junctional, cortical and focal adhesion forces. The observed behavior was similar with the shallow and sharp stiffness gradients compared to the interface gradients in equal distances from the manipulated cell (Supplementary Fig. S3). These gradients also produced similar relative peaks in the focal adhesion forces; however, the wider the stiffness gradient, the more spread out and lower the peak was.

Together, our results suggest that cells situated on a soft island move less and are subjected to larger cortical, junctional and focal adhesion forces if one cell experiences a substantial deformation or movement.

#### From a Stiff to a Soft Substrate

**Fig. 7:**
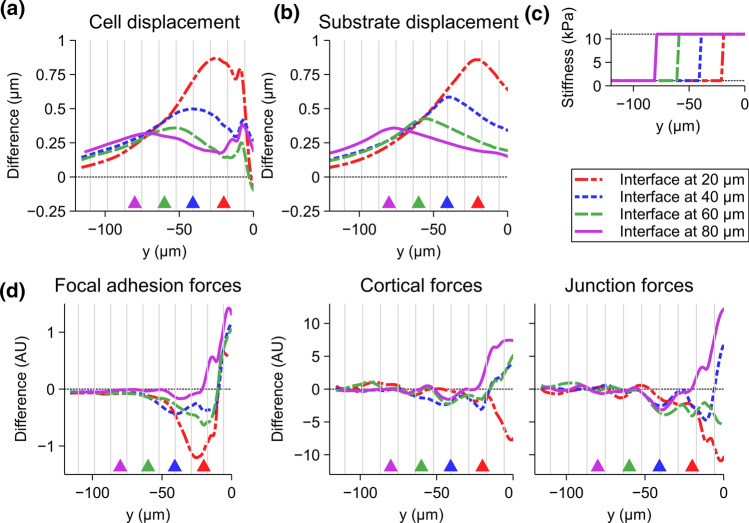
The spread of displacement and forces from stiff substrate region to a soft region. The manipulated cell is positioned at the initial pipette position [coordinates (0, 0)]. The panels show the differences in vertical cell displacement and focal adhesion, cortical, and junction force transmissions caused by an interface gradient. The absolute difference in **a** cell and **b** substrate displacement compared to the uniform 11-kPa displacement for stiffness interface gradients at 20, 40, 60, and 80 $\upmu$m. **c** The stiffness interfaces for displacement and forces shown in **a**–**b** and **d**. **d** The differences in focal adhesion, cortical, and junction forces for the stiffness interfaces compared to the average cell forces in the corresponding position with 11-kPa substrate. The magnitudes of the displacement and forces are shown in Supplementary Fig. S6. The vertical striping shows the positions of cell boundaries for average sized cells and the positions of the interfaces are shown with the arrowheads of corresponding colors at the bottom of each figure. For each case, n = 15. *AU* arbitrary unit. See Fig. 6b for explanation of how the absolute and relative differences were calculated

Next, we wanted to investigate how the force transduction is altered in the opposite case: When the manipulated cell is within the stiff region of the substrate and the stiffness decreases at some distance (Fig 7c). Thus, we simulated the 20-$\upmu$m micromanipulation of a single cell with the stiffness profiles mirroring those in the previous section. Here, the cell and substrate movements were increased in comparison to the uniform 11-kPa substrate (Fig. 7a, b; Supplementary Fig. S5). The cells moved more around the stiffness interfaces (Fig. 7a), with the larger increase in displacement the closer the interface was to the manipulated cell. Interestingly, the displacement was increased well before the interface itself, also for those farther away from the initial pipette position. The general behavior of the difference in the substrate displacement was similar to that of the cells but with slightly higher peak values (Fig. 7b).

The changes in the forces compared to the uniform stiffness were minor at distance of more than 50 $\upmu$m from the manipulated cell independent of the interface location (Fig. 7d). The differences in the junction and cortical forces were negative when the interface was close to the manipulated cell and became positive as the interface moved farther away (Fig. 7d). On the other hand, focal adhesion forces were increased near the initial pipette position but showed generally reduced values at longer distances. Furthermore, following the interfaces at 20 and 40 $\upmu$m, there were inverse peaks in these forces compared to the uniform substrate (Fig. 7d), which were not as clearly visible with the farther interfaces. The data indicates that the single-cell movement within an island of stiff substrate causes larger deformations in the neighboring cells than on a uniform stiffness.

Again, the differences in the cell displacements and forces were similar with the shallow and sharp gradients compared to the interface gradients in similar locations (Supplementary Fig. S5). The reduced focal adhesion forces were also visible following the decrease in stiffness similar to those in Fig. 7d.

### The Effect of Substrate Stiffness on Small Changes in Cell Shapes

**Fig. 8:**
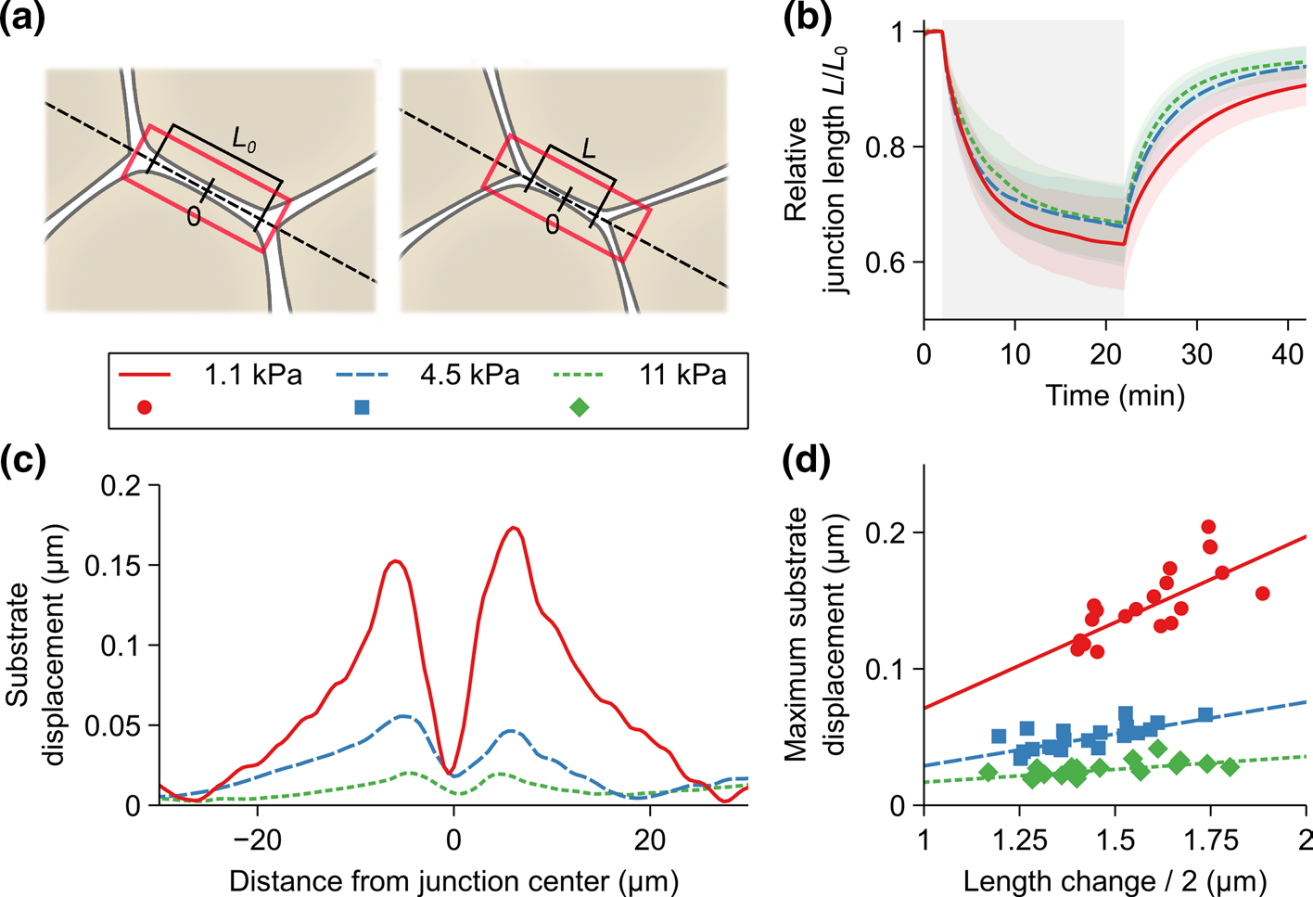
Reduction of the cell–cell junction length and substrate displacement during optogenetic activation. **a** To simulate the increased cortical contractility due to light activation, a rectangular area enclosing the cell–cell junctions between two cells (the red rectangle) was selected for activation. We then monitored the change in the junction length (*L*) from the initial state ($L_{0}$) as a function of time. **b** We used a single activation of 20 min (gray area between 2 and 22 min) and calculated the relative junction length ($L/L_{0}$) during this activation and the following relaxation. This was done for the stiffnesses 1.1, 4.5, and 11 kPa. For each stiffness, n = 20. The shaded area indicates standard deviation. **c** Representative plots of the displacement of the substrate with the three stiffnesses along the axis of the junctions (the dashed line in **a**) in relation to the junction center point (indicated in **a**). **d** Maximum substrate displacement as a function of half of the change in the junction length for each simulation for the three stiffnesses. The maximum substrate displacement was calculated as mean of the peaks on each side of the junction center point shown in **c**. The lines show linear fit for each set of points

Our computational model indicated that the substrate stiffness and especially stiffness gradients influence the strain distribution after a large single-cell movement. We also investigated how small changes in cell–cell junctions are transmitted to the surrounding substrate in the timescale of minutes. To correlate the simulations to experimental data, we simulated an optogenetic experiment, where actomyosin contractility is increased by light activation. Therefore, we implemented optogenetic activation into our modeling platform based on the experimental and theoretical work by [56]. We obtained the model parameters either directly from Staddon et al. or by fitting as described in the Supplementary Text. We did not consider the strain-based remodeling of the cortical tension since we wanted to concentrate on the effect of the substrate stiffness on the local movement of cell boundaries, and the tension remodeling primarily affects the permanently reduced junction length after the optogenetic activation [56]. We also allowed the remodeling of the cell structures in these simulations due to the long experiment duration compared to the micromanipulation. We increased the contractility of cell vertices forming the junctions between two cells to observe how the length of this section of junctions reduced following the activation (Fig. 8a and Supplementary Video S3). We ran the simulations on substrates with uniform stiffnesses of 1.1, 4.5, and 11 kPa.

During the simulated 20-min activation, the increased cortical contractility shortened the junction length the most with the softest 1.1-kPa substrate with the final relative length of around 0.63 ± 0.08 (mean ± SD, Fig. 8b). The relative junction length was reduced to similar values of 0.66 ± 0.07 and 0.67 ± 0.07 for 4.5 and 11 kPa, respectively (Fig. 8b). However, the initial length reduction was faster with the 4.5-kPa substrate than the 11-kPa, with a similar slope to the 1.1-kPa substrate.

Next, we studied how the shortening of the cell–cell junction deformed the substrate. We defined the maximum displacement of the substrate field between the time points before the activation (time = 2 min) and the end of the activation (time = 22 min) along a line defined by the activated section of junctions between the cells (the dashed line in Fig. 8a) for each simulation. Figure 8c shows a representative displacement plot for each stiffness centered on the junction center point. To quantify the results, we took the average displacement peak values on each side of the center point. We plotted them as a function of half of the change in the junction length between the two time points for each simulation (Fig. 8d). Half of the length change was used since one of the displacement peaks in Fig. 8b resulted from half of the total junction length reduction. To compare these relative displacements with those from the micromanipulations, we calculated the mean maximum substrate displacement in relation to the corresponding half of the junction length reductions. The obtained values were 0.093, 0.035, and 0.018 $\upmu$m respectively for 1.1-, 4.5-, and 11-kPa substrates, which were considerably smaller than the corresponding values in the 30-$\upmu$m micromanipulations, indicating a nonlinear relationship between the cell and substrate displacements.

The simulation results show that the substrate stiffness has only a minor direct effect on the small cellular morphological changes. In addition, these small changes in the cell morphology could not deform the substrates at a visible level, especially with the higher stiffnesses (Fig. 8c).

## Discussion

The role of mechanical forces in cellular communication and in the regulation of cell functions has been widely accepted [29, 34, 42]. Moreover, the stiffness of the cellular microenvironment is known to affect the behavior and properties of the cells, and an increase in the stiffness has been linked to many diseases. Most notably, in tumor formation and cancer progression, the ECM stiffness increases [5, 13], possibly affecting the transmission of mechanical strain between cells. Tightly packed epithelial monolayers on deformable substrates form an excellent platform for studying how forces are transmitted between cells and what is the effect of substrate stiffness in this process. Furthermore, 80–90% of cancers originate from epithelial cells, making it relevant model also for cancer development [3, 26]. Epithelia have been used to study active force transmission between cells over long timescales [24, 37, 62], however, the way that elastic forces are rapidly transmitted within epithelial monolayer has not been fully addressed. We developed a computational model to describe the fast transmission of passive, elastic forces in the cell monolayer on deformable substrates based on our own experimental data and from the literature.

We first studied how a substrate with a uniform stiffness affects the displacement of cells, and therefore the transmission of elastic forces, following an exogenous 30-$\upmu$m movement of a single cell over 1 s. Logically, both the cell and the substrate displacements spanned over longer distances the softer the substrate. The high cell displacement perpendicular to the pipette movement observed with the soft 1.1-kPa substrate can be explained by the stiffness of the MDCK monolayer, that has been reported to be between 1 and 5 kPa, when measured with atomic force microscopy [20, 40, 43]. This means that the stiffness of the epithelial monolayer has a similar or slightly higher stiffness than the softest substrate and, therefore, can more readily displace the substrate than the monolayers on the stiffer substrates. We observed only minor differences between the perpendicular cell displacements on the stiffer substrates, suggesting that the effects begin to saturate as stiffness increases. Therefore, having a substrate stiffer than 35 kPa would most likely have no further effect on the cell displacements. We want to highlight that these stiffnesses are slightly higher than reported for many healthy tissues or tumors in 3D in vivo environment, where stiffness can be less than 1 kPa [19]. However, the used 2D epithelial system still allowed us to probe how substrate stiffness or stiffness gradient in the range of physiological conditions affects the force transduction within the epithelium.

The observed difference between the maximum cell and substrate displacements can be explained by the different movements of the apical and basal surfaces of the cell. Both our imaging of the mEmerald-Occludin-expressing MDCK cells and our computational model describe the cells by their apically located adherens junctions. The displacement of the basal substrate-binding cell membrane is more directly conjugated to the substrate displacement. This suggests that the cell shape in the apico-basal axis is heavily deformed, especially near the micromanipulated cell. Furthermore, the cell and substrate displacements varied more in the experimental results compared to the computational simulations. Therefore, the variability in the epithelial morphology—i.e., the cell sizes and shapes—was not enough to explain the experimental displacement variability, and our simulation results thus only reflect an average epithelium. In reality, the mechanical properties are more heterogeneous within the monolayer.

We used our computational model to study the cortical, cell–cell, and cell–substrate forces during the micromanipulation. It is noteworthy to mention that the focal adhesion forces describe both the tension in the focal adhesions and the apico-basal elasticity of the cell itself. As expected, we found all of these forces to increase with the substrate stiffness. As the cell monolayer was always subjected to the same external strain, in the stiffer environment the smaller substrate deformation meant that the cells were subjected to a larger portion of this strain. This led to a larger cell deformation in the apical plane and higher cell strain in the cells’ apico-basal axis. These changes corresponded to the increases in the cortical and focal adhesion forces, respectively. Importantly, for the apical cell deformation to occur, the next cell opposite to the incoming strain must resist movement or deformation. If the next cell can be readily displaced, less of the mechanical energy goes to cell deformation as it is easier to transmit it onward. Therefore, the cortical and junction forces depend on each other since higher resistance against deformation in the cortex leads to higher junction forces as less of the mechanical energy is absorbed by the cell cortex. However, the differences in the cortical and junction forces between the stiffnesses disappeared beyond the distance of 50 $\upmu$m, indicating that the bulk of the mechanical energy is absorbed closer to the manipulated cell on the stiff substrates.

The focal adhesion forces remained higher for the cells on stiffer substrates due to the larger difference between the cell and substrate displacements and the higher focal adhesion strength. Similar results were found by [21] in the developing *Drosophila* embryos, as they showed that the amount, and thus the strength, of basal cell–ECM adhesions was inversely correlated with the displacement of the apical surface of the cell. They hypothesized that the increased apical displacement was the result of more efficient apical force transmission. On the other hand, our results indicated higher forces transmitted between cells with stronger focal adhesions and smaller apical displacements in our elastic system with an exogenous mechanical stimulus.

We also studied how stiffness interfaces and gradients in the substrate affect the transmission of forces in the epithelium. In the simulations the manipulated cell was either on soft island (1.1 kPa), which was surrounded with a stiffer substrate (11 kPa), or vice versa. The material interface and thus the stiffness changes situated 20–80 $\upmu$m, from the manipulated cell. Stiffness interfaces and gradients, for example between healthy and tumor stroma, have been shown to affect the integrity of endothelial monolayers and impact their behavior over distances of more than a hundred micrometers [65]. Similarly, we found that the cell displacement was affected, even if the stiffness interface was 80 $\upmu$m away from the manipulated cell. The cortical and junction forces in cells situated on a soft island increased, indicating that they experience more force than on a substrate with a uniform soft stiffness. However, after the soft-to-stiff interface the increase in these forces was rapidly diminished. The simulations also showed that the cell and substrate displacement changes had a local minimum in the vicinity of the interface, when compared to the uniformly soft substrate. This is most likely because the cells on the stiff region were more difficult to displace due to their stronger binding to the substrate and the stiffer substrate itself. These data indicate that the cells on the softer environment are mechanically more isolated from the surrounding cells growing on a stiffer substrate due to the soft-to-stiff rigidity interface.

The situation was opposed in the case of manipulated cell being on a rigid island, surrounded by softer substrate. Now the cell and substrate displacements were larger beyond the stiff-to-soft interface when compared to homogeneously stiff substrate. Junctional and cortical forces were less affected and focal adhesion forces showed overall reduced force near the manipulated cell. Thus, the stiffer island did not lead into mechanically isolated cells, but especially the cell and material displacement or deformation was readily transmitted for long distances from the stiff-to-soft interface into the softer environment. Interestingly, we observed only minor differences in the cell displacement or forces in relation to the slope of the increase or decrease in stiffness. Therefore, whether the change in stiffness occurs within 2 or 50 $\upmu$m, the main factors that affected the cells were the change in stiffness and its distance from the manipulated cell.

Furthermore, we used our model to study how the substrate stiffness affected the small local changes in cell shape by implementing an optogenetic control of myosin activation. The results suggested that the substrate stiffness has only a minor effect on the small changes in the cell–cell junction elastic behavior. This can be explained by the separation of the apical and basal surfaces, as small morphological changes in the apical side were not greatly restricted by the substrate binding in the basal side of the cells. The observed difference in the relative substrate and cell displacement between the optogenetic and micromanipulation simulations showed that the displacement of the apical side of the cells has to be extensive enough to visibly deform the substrate due to the compliance provided by the cells’ apico-basal axis.

**Fig. 9:**
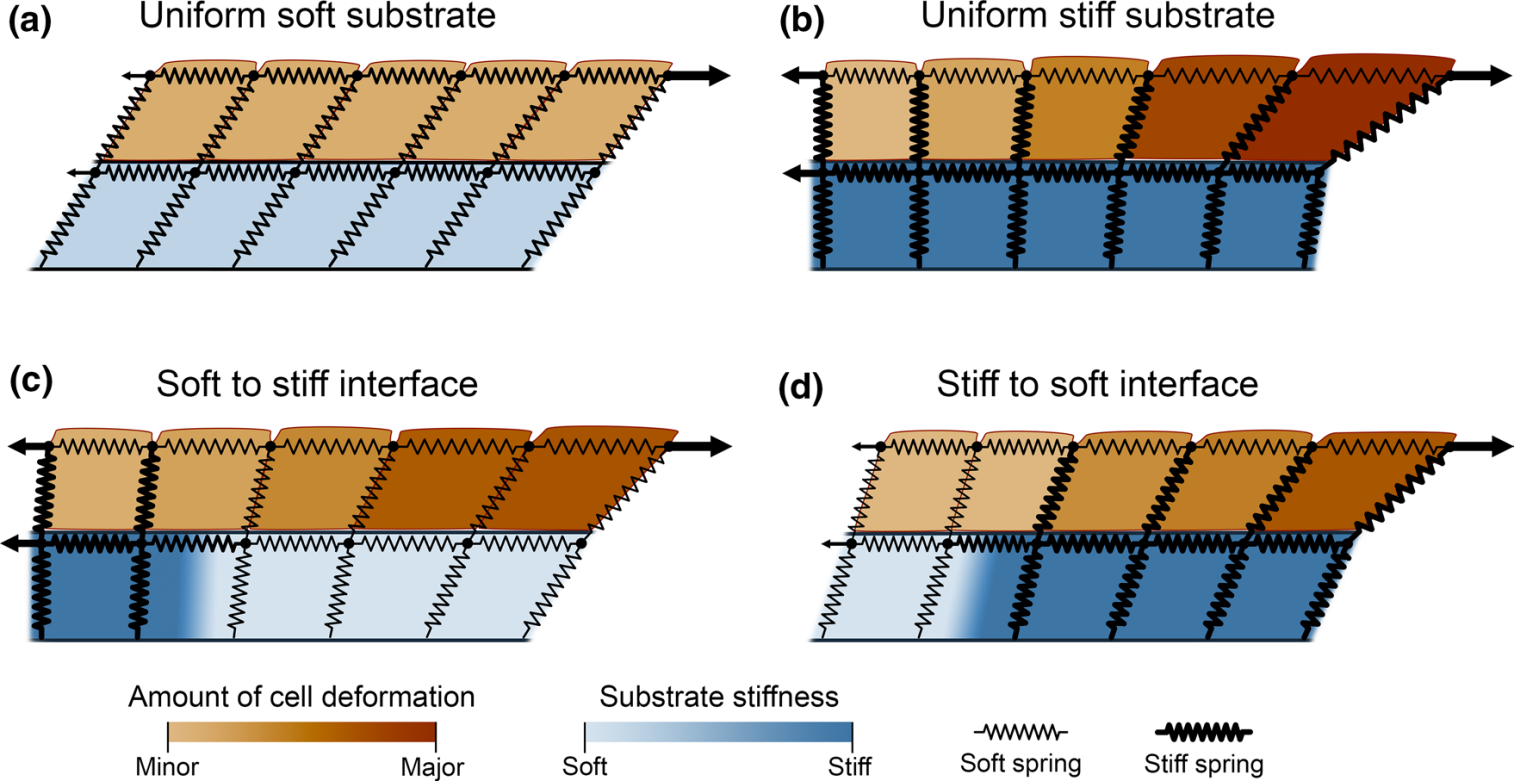
A 2D crosscut abstraction of the effect of substrate stiffness and stiffness interfaces on cell displacement and deformation upon mechanical stimulus. **a** On a uniform soft substrate, the cells are easily moved and do not substantially deform since the neighboring cells can also be readily moved. **b** A uniform stiff substrate is challenging to deform, leading to limited cell displacement, that is further limited by the stronger focal adhesion forces compared to the soft substrate. This leads to large deformation of the cells due to the limited ability of the neighboring cells to move. **c** When there is an increase in stiffness at a distance, the movement of cells on the stiff side of the stiffness interface is suppressed by the limited movement of the cells. This leads to more deformation of the cells on soft side of the interface compared to the uniform soft substrate. **d** With a decrease in stiffness at a distance, the cells on the soft substrate can readily move, enabling large cell movement also on the stiff substrate and thus less cell deformation compared to the uniform stiff substrate. The lengths in the figures are not to scale

The factors affecting the displacement and deformation of cells following some external force can be summarized as follows (Fig. 9). First, the stiffness of the substrate to which the cell is attached—together with the strength of this attachment—modulates the apical displacement. The second factor is the ability of the neighboring cells to be displaced or deformed. The cells move easily on a soft uniform substrate, and therefore, the cell deformations are limited as it is easier to transmit the strain to the neighboring cells. On the other hand, the cell positions are more fixed on a stiffer substrate, making their movement along with the substrate more difficult, which leads to increased cell deformation. Furthermore, for example in the case with a stiffness increase opposite to the incoming force, the displacement of a cell in the soft region is limited by the less movable cells in the stiff region, leading to larger apical deformation. This further indicates that the information of the changes in stiffness farther away may be transmitted as forces between the cells.

This transmission of the information of the mechanical properties of the substrate is highly dependent on the cells’ resistance against deformation. If an external force can readily deform and strain the cell’s apical surface, less mechanical energy is left to be transmitted to the neighboring cells. Prestress in the cytoskeleton leads to resistance against cell deformation and has been shown to enable longer distance mechanical communication within cells compared to homogeneous solids [27, 39, 68]. It seems to be important also for long-range mechanical signaling within an epithelial monolayer. A similar effect is seen with fibrous substrates since separated cells can communicate via the substrate over long distances [28, 36, 45].

The developed computational model, together with the graphical user interface, form a platform to complement the existing cell-based methods, by, to our knowledge, for the first time describing in detail the mechanics of the epithelial monolayer in combination with those of the underlying deformable substrate. The chosen method enables the detailed description of the cell shape as well as the inclusion of various subcellular structures. Compared to the common vertex model, our approach describes the cells and their interactions in more detail while being computationally heavier. Furthermore, the model provides an additional level of complexity and dynamics compared to the previous closed-polygon-based models, e.g., [58], since the cells are allowed to divide during growth and change their size and perimeter, and the junctions between the cells are allowed to remodel. A similar tissue model to ours was recently published [10], building on the work by Tamulonis and coworkers with more dynamic cell functions and properties, but lacking the deformable substrate. The few models that describe the substrate have not considered the effect of its mechanical properties in relation to the epithelial mechanics [30, 50]. In addition to the simulations presented here, the developed computational platform enables the description of further typical mechanical experiments conducted with epithelia, e.g., lateral substrate compression or stretching. The modeling concepts could also be model the mechanics in other tissue, such as mechanically active cardiac tissue.

While our modeling approach generally describes the system formed by the epithelial monolayer and the substrate well, there are limitations. First, the model cannot correctly describe the force transmission perpendicular to the micromanipulation on soft substrates, which can be explained by the rotation of the cell–cell junction interactions in relation to the cell membranes. Secondly, based on the slightly higher cell displacement at longer distances with the stiffer substrate predicted by the model compared to the experimental data, it seems that the linear springs’ ability to describe the passive cell mechanics might be limited to cases with smaller strains. Third, the substrate model was unable to describe the behavior of hydrogels with a higher stiffness. The parameters describing the cell mechanics, excluding those of the focal adhesions, were assumed to be independent of the substrate stiffness. While cell stiffness has been previously shown to mimic that of the substrate [55, 60], it was recently reported that these measurements may underestimate the cell stiffness on soft substrates and that the cell stiffness is largely independent of that of the substrate [47]. Furthermore, the description of the focal adhesion forces is challenging since they included both the focal adhesions themselves and the stiffness of the cell in the apico-basal direction. Separating these two components into their own forces could better describe the mechanics. All these limitations most likely have a negligible effect on the main conclusions of the study.

In summary, results from our experimental and computational work on elastic epithelial biomechanics suggest that the mechanical properties of the substrate have a significant effect on the distance over which elastic forces spread in the epithelial monolayer over short timescales. Furthermore, we found that the substrate stiffness gradients influence the strain distribution in epithelial monolayers. The result indicates that, for example, the altered stiffness of tumors can affect how the mechanical strain is transmitted outside of the tumor itself, or vice versa. This can have a direct effect on neighboring cells via e.g., stretch activable mechanosensitive ion channels (e.g., Piezo1 [22]). However, further studies are needed to better understand the role of each component in this phenomenon. The computational cell-based model presented here forms a valuable platform for futures studies on epithelial mechanics. In the future, the model would benefit from adding more viscous behavior and active biomechanics, such as the tension remodeling described by [56] and the inclusion of the cell nuclei. The latter would also allow the study of the forces felt by the nucleus and thus their possible role in regulating gene expression [29, 35]. Furthermore, since the more fibrous nature of the natural ECM has been shown to transmit forces over longer distances [28, 36, 45], it would be interesting to study the ability of a fibrous substrate to spread strain in the epithelial monolayer.

## Materials and Methods

### Cell Maintenance and Establishment of MDCK mEmerald-Occludin-Expressing Cells

We used MDCK II (ATCC CCL-34) cells as an in vitro epithelial model tissue. The cells were cultivated in standard conditions in a humidified cell incubator (+ 37 $^{\circ}$C, 5% CO$_{2}$) and maintained in Modified Eagle’s medium (#51200046, Thermo Fisher Scientific, Waltham, MA, USA) supplemented with 1% (vol/vol) antibiotic (#15140122, Thermo Fisher Scientific) and 10% fetal bovine serum (#10500064, Thermo Fisher Scientific). The MDCK cells used in the micromanipulation experiments stably expressed mEmerald-Occludin to highlight the cell–cell junctions with fluorescence. mEmerald-Occludin was a gift from Michael Davidson (Addgene plasmid #54212; http://n2t.net/addgene:54212; RRID: Addgene_54212). The MDCK mEmeral-Occludin cell line was established by first transfecting the MDCK cells with the mEmerald-Occludin plasmid using Neon Transfection system (Thermo Fisher, Waltham, USA). One day after the transfection, we started the positive cell selection with a medium where we replaced P/S with 0.75 mg/ml G418 antibiotic (#Gnl-41-01, Thermo Fisher Scientific). We picked positive colonies approximately 2 weeks later using a fluorescent microscope situated in the sterile cell culture hood. The MDCK mEmerald-Occludin cells were maintained in Modified Eagle’s medium (#51200046, Thermo Fisher Scientific, Waltham, MA, USA) supplemented with 0.25 mg/ml G418 antibiotic (#Gnl-41-01, Thermo Fisher Scientific) and 10% fetal bovine serum (#10500064, Thermo Fisher Scientific).

### Polyacrylamide Hydrogels and Cell Culturing

The PAA hydrogels were cast on 18 $\times$ 18 mm glass coverslips. First, coverslips were cleaned by immersing them in 2% Helmanex for 1 h in + 60 °C, followed by washes with excess water and ethanol. The coverslips were then let to dry in a fume hood or dried with a nitrogen stream. The cleaned coverslips were stored in a desiccator.

Before gel casting, the surfaces of the coverslips were amino-modified with 3-(Trimethoxysilyl)propyl methacrylate (#M6514, Sigma-Aldrich, Saint-Louis, USA) to allow firm gel attachment. The 3-(Trimethoxysilyl)propyl methacrylate and glacial acetic acid were mixed with 95% ethanol yielding final concentrations of 0.3% (vol/vol) and 5% (vol/vol), respectively. The solution was let to react with a glass coverslip for 3 min at RT. Next, the coverslips were washed with excess ethanol and air-dried in a fume hood. The activated coverslips were stored in a desiccator.

The different gel rigidities were achieved by mixing different ratios of gel precursors acrylamide (AA, stock 40%, #1610140, Bio-Rad Laboratories, Hercules, USA) and bis-acrylamide (Bis, stock 2%, #1610142, Bio-Rad Laboratories, Hercules, USA) with PBS in 15 ml falcon tube [64]. The following mixing ratios were used: for 1.1 kPa gel final concentrations of AA and Bis were 3% and 0.10%, respectively; for 4.5 kPa 5% and 0.15%; for 11 kPa 10% and 0.10%; and for 35 kPa 10% and 0.30%. The gel precursor solution was then degassed with a vacuum. Next, 2 ml of this solution was pipetted into a new 15 ml falcon tube, and 2% (vol/vol) fluorescent beads (0.2 $\upmu$m diameter, red fluorescent, #F8810, Thermo-Fisher, Waltham, USA) were added and mixed without bubble formation.

The gel polymerization was initiated by adding TEMED (#1610800, Bio-Rad Laboratories, Hercules, USA) and APS [10% (weight/vol) stock solution in PBS, #A3678-100G, Merck, Kenilworth, USA] to a concentration of 0.2% (vol/vol) and 1% (vol/vol). The gel was mixed by tilting the tube 3–5 times, and immediately afterward, 13 $\upmu$l of gel solution was pipetted on an activated coverslip. Next, 13 mm cleaned but unactivated coverslip was carefully placed on top, sandwiching the polymerizing gel between the two coverslips. Gels were allowed to polymerize for 45 min in a moist chamber at RT. After polymerization, the gel-coverslip sandwiches were placed on 6-well plates, immersed in PBS, and kept o/n at + 4 °C. On the following day, the 13 mm coverslips were carefully removed using a sharp scalpel, yielding approximately 100 $\upmu$m thick PAA gels on 18 $\times$ 18 mm coverslips.

Finally, the gels were coated with collagen-I to facilitate cell adhesion and growth. The coating was conducted by using 3,4-Dihydroxy-l-phenylalanine (L-DOPA) (#D9628, Sigma-Aldrich) according to [69]. L-DOPA was dissolved in the dark to 10 mM Tris buffer, pH 10, with a final concentration of 2 mg/ml. The gel samples were incubated with L-DOPA solution for 30 min at RT in the dark. Next, the samples were washed twice with PBS and collagen-I in concentration of 50 $\upmu$g/ml in PBS was added on top of the gel and incubated for 1 h at RT. Finally, the cells were washed twice with PBS, and cell seeding was conducted immediately.

MDCK II cells stably expressing mEmerald-Occludin were maintained in 75 cm^2^ cell culture flasks. The protein-coated gels were placed on sterile 6-well plates with PBS and sterilized in the laminar under UV light for 15 min. The cells were trypsinated and suspended into 10 ml of cell culture medium, and 100 $\upmu$l of the cell suspension was then pipetted on each gel, and 2 ml of medium was added to the well. Cells were cultured for 7 days prior to the micromanipulation experiments.

### Imaging and Micromanipulation

We imaged the epithelial mechanics during micromanipulation using Nikon FN1 upright microscope (Nikon Europe BV, Amsterdam, Netherlands) with CFI Apo 40x/0.8 water-dipping objective. The mEmerald-Occludin was excited with 470 nm LED and beads with 580 nm LED from pE-4000 light source (CoolLED Ltd., Andover, UK). The system was equipped with W-VIEW GEMINI image splitting optics (Hamamatsu, Sunayama-cho, Japan), allowing simultaneous capturing of mEmerald-Occludin and fluorescent bead channels. The camera used in imaging was sCMOS ORCA-Flash 4.0 v2 (Hamamatsu, Sunayama-cho, Japan), which yielded an image pixel size of 330 nm. The used exposure time was 50 ms. During timelapse imaging, the frame rate was 13.4 frames/s.

Micromanipulation was conducted using uMp-3 triple-axis micromanipulator with uMp-TSC controller and uMp-RW3 rotary wheel interface (Sensapex, Oulu, Finland). The cells were manipulated by using a glass micropipette, similar to those used in patch-clamp recordings. The pipettes were constructed with P-1000 micropipette puller (Sutter Instruments, Novato, USA) and afterward closed with micro forge MF-830 (Narishige, Tokyo, Japan), yielding blunt pipette tips.

In the epithelium micromanipulation, the micropipette was brought in contact with a cell. The contact was visible in the microscopy images as a small indentation of the cell membrane. The pipette was then lowered between a few hundred nanometers to 1 $\upmu$m to capture the cell–cell junctions when the pipette was moved. We started timelapse imaging and subsequently moved the micropipette 30 $\upmu$m with a speed of 30 $\upmu$m/s perpendicular to the pipette orientation. The movement was controlled via uMx Software (Sensapex, Oulu, Finland) using its macro commands. This yielded rapid mechanical manipulation of the cell and a movement of approximately 15 $\upmu$m of its center.

For immunostaining FAKs, MDCK II cells were cultured on PAA hydrogels with stiffnesses of 1.1, 4.5, 11, and 35 kPa for 7 days. Cells were fixed with 4% PFA (v/v, #157-8, Electron Microscopy Sciences, UK) for 10 min and rinsed three times with 1 $\times$ PBS. Permeabilization was done in 0.5% Triton-X-100 (v/v, #T8787, Sigma-Aldrich, US) and 3% BSA (w/v, #P06-139210, PAN-Biotech, US) in 1 $\times$ PBS for 10 min RT. Blocking was done in 3% BSA in 1 $\times$ PBS for 1 h at RT. Anti-FAK [1:100, anti-FAK phospho Y397 (EP2160Y), ab81298, Abcam, UK] primary antibody incubation was done in 3% BSA in 1 $\times$ PBS at RT. Samples were washed three times 10 min, first in permeabilization buffer, then in 1 $\times$ PBS, and last in permeabilization buffer. Anti-rabbit Alexa 488 (1:200, #A-11008, Invitrogen, USA) secondary antibody incubation was done in 3% BSA in 1 $\times$ PBS at RT with ATTO 565 Phalloidin (1:100, ATTO-TEC, Germany). Samples were washed three times in 1 $\times$ PBS for 10 min and stored in 1 $\times$ PBS at + 4 °C. Samples were imaged using Nikon FN1 upright microscope with 40x/0.8 water-dipping objective, as described above. Image pixel size was 108.3 nm. The Alexa 488 was excited with 470 nm LED and Atto 565 with 550 nm LED from pE-4000 light source (CoolLED Ltd., Andover, UK).

### Data Analysis

The experimental imaging data before and after the micromanipulation pipette movement was initially segmented using the Trainable Weka Segmentation [2] plugin of ImageJ Fiji [51]. We randomly selected six images from the imaging data set to train the classifier to segment the cells based on the mEmerald-Occludin data to obtain the cell boundaries. Next, the probability maps were converted to binary masks using Find maxima and then skeletonized. We manually fixed any errors in the skeleton images based on comparison with the original images. Finally, BioVoxxel Toolbox’s Extended Particle Analyzer [4] was used to analyze the final segmented binary images. We tracked the movement of the cell centers between the segmented images before and after the pipette movements using a custom, semi-automated MATLAB script (R2020b, The MathWorks, Inc., Natick, Massachusetts). The movement data was then used to interpolate the cell movement in relation to the original pipette position to obtain a cell movement map. Finally, we averaged the movement maps over the data from each gel stiffness.

We analyzed the gel deformation based on the fluorescent microbead data using Fiji’s PIV analysis plugin between the images before and after the micromanipulation. Similar to the cell data, the gel deformation maps were centered on the original pipette position and averaged over the same stiffnesses.

### Computational Modeling

A detailed description of the model, the fitting, and the simulations are available in the Supplementary Text. In our model, the epithelium was described as a two-dimensional monolayer, with each cell represented by a closed polygon (Fig. 3a). The model was based mainly on the boundary-based model by [58] but borrowed features from the vertex models [18, 50]. Cell structures and processes were incorporated into the model as forces affecting the polygon vertices. These include cortical actomyosin, cell–cell junction dynamics, intracellular pressure, cell division, focal adhesions, and membrane elasticity. Some of these forces are depicted in Fig. 3c. Furthermore, the cortical dynamics included the actomyosin prestress, described by a constant force component and a perimeter-dependent tension component. The number of the cell vertices was not static: new vertices were added to divide long membrane sections, and vertices were removed if a section between two vertices became too short. In addition, the cell–cell junctions were dynamic and constantly remodeled during the simulation.

The top surface of the underlying substrate was represented by a two-dimensional triangular grid of points (Fig. 3a, b). As with the cells, the substrate mechanics were represented by forces acting on the grid points. The forces were related to the internal mechanics of the substrate as well as to the focal adhesions.

Equation of motion was used to evolve the model system during the simulation. The system was assumed to be overdamped, enabling the omission of inertial effects. This simplification is commonly done as the importance of inertia is small in biological systems [17, 18]. The movement of cell vertex *i* and substrate point *m* were calculated as

1 $$\eta \frac{d{\textbf{r}}_{i}}{dt} = {\textbf{F}}_{i,tot},$$

2 $$\eta \frac{d{\textbf{s}}_{m}}{dt} = {\textbf{F}}_{m,tot},$$

where $\eta$ is the dampening coefficient (kg/s), $r_{i}$ is the position of the cell vertex *i* (m), $s_{m}$ is the position of the substrate point *m* (m), *t* is time (s), and $F_{i,tot}$ is the total force acting on cell vertex *i* (N) and $F_{m,tot}$ that on the substrate point *m* (N). The total force for each cell vertex *i* was calculated as the sum of these component forces:

3 $$\begin{aligned} {\textbf{F}}_{i,tot} =&{\textbf{F}}_{i,cort} + {\textbf{F}}_{i,junc} + {\textbf{F}}_{i,area} + {\textbf{F}}_{i,div}\\&\quad + {\textbf{F}}_{i,fa} + {\textbf{F}}_{i,mem} + {\textbf{F}}_{i,cont} + {\textbf{F}}_{i,edge}, \end{aligned}$$

where ${\textbf{F}}_{i,cort}$ is the cortical actomyosin force, ${\textbf{F}}_{i,junc}$ the cell–cell junction force, ${\textbf{F}}_{i,area}$ the area force that describes the internal pressure, ${\textbf{F}}_{i,div}$ the division force, ${\textbf{F}}_{i,fa}$ the focal adhesion force, ${\textbf{F}}_{i,mem}$ the membrane force, ${\textbf{F}}_{i,cont}$ the contact force, and ${\textbf{F}}_{i,edge}$ is the edge force. The last two forces had an auxiliary role: the contact force described contact between cells and prevented cell overlap, and the edge force described the continuity of the epithelium outside the simulated area.

The substrate mechanics were divided into three forces: a central force between neighboring points, a repulsive force between a point and the connection between two of its neighbors, and a restorative force that sought to move a point to its original location. The second force was included to prevent the collapse of the substrate during large deformations [11], and the third to describe the fact that the substrate was attached to rigid glass at its bottom surface in our experiments. Furthermore, a fourth force component was included to depict the cell–substrate connection via the focal adhesions. Now, the total force affecting each substrate point was calculated as

4 $${\textbf{F}}_{m,tot} = {\textbf{F}}_{m,cent} + {\textbf{F}}_{m,rep} + {\textbf{F}}_{m,rest} + {\textbf{F}}_{m,fa},$$

where ${\textbf{F}}_{m,cent}$ is the central force between closest neighboring points, ${\textbf{F}}_{m,rep}$ is the repulsive force to prevent material collapse, ${\textbf{F}}_{m,rest}$ is a restorative force, and ${\textbf{F}}_{m,fa}$ is the force from the focal adhesions.

The model was used to simulate epithelial growth and the tissue response to two different mechanical stimuli: (1) pointlike micromanipulation in a short time scale and (2) a local increase in actomyosin tension by optogenetics over a longer time scale.

We used the model to grow epithelia from a single cell (Fig. 8d and Supplementary Video S4) to produce epithelium of sufficient size without the substrate. The randomness in the tissue was produced by normally distributed times between divisions and cell area distribution based on our in vitro MDCK cell data. The size of the grown epithelium was chosen based on the assumed effect of each mechanical stimulus to minimize the impact of the tissue edges. Following the growth, the epithelia were given time to relax without division to remove any stresses. Next, the grown epithelia were placed on the substrate, and the focal adhesions were defined between the two.

Corresponding to our micromanipulation experiments, we moved a single cell by an external force with a known speed over a distance. Since we wanted to describe the elastic behavior, we prohibited any changes in the number of cell vertices and cell-cell junctions in these simulations, justified by the short time scale of these measurements. The values of the model parameters governing the cell mechanics were fitted using our in vitro micromanipulation data with the uniform 1.1-, 4.5-, and 11-kPa substrates by iteratively changing the parameter values and comparing the cell center and substrate displacements between the experimental data and simulations results. The fitted model was then used to study the force distribution on the uniform substrates and those with stiffness interfaces and gradients. The interfaces and the gradients were defined along the direction of the virtual pipette movement and characterized by the gradient slope and distance from the initial pipette position.

In the optogenetic activation simulations, the contractility of the cortex in a section between two randomly chosen cells was increased to describe the experimental myosin activation. This was done by increasing the value of cortical tension constants for the cortical forces within the activation region. The parameters for these simulations were obtained from [56] and by fitting our model to their data. The model was solved using either 2nd or 4th order Runge–Kutta methods with variable time steps. During the growth simulations when the substrate was excluded, 2nd order Runge–Kutta was used since it was sufficiently accurate. Since these simulations lacked the substrate and did not consider that the epithelium continues beyond the existing cells, the focal adhesion and the cell edge forces were omitted. During the simulations that included the substrate, 4th order Runge–Kutta was used.

The model is implemented in MATLAB, where we also created a graphical user interface for the model platform. The model code is available in GitHub (https://github.com/atervn/epimech) and achieved in Zenodo [61].

### Supplementary Information

Below is the link to the electronic supplementary material.

(PDF 7435 kb)

(PDF 3048 kb)

(MP4 44949 kb)

(MP4 15047 kb)

(MP4 7067 kb)

(MP4 4310 kb)

## Data Availability

Data available upon request.

## Code Availability

The code is available in GitHub (https://github.com/atervn/epimech) and achieved in Zenodo [61].
