# Supplementary material for: The Effect of Substrate Stiffness on Elastic Force Transmission in the Epithelial Monolayers over Short Timescales
Source: Cell Mol Bioeng. 2023 Jul 13;16(5-6):475–95. doi: 10.1007/s12195-023-00772-0 (PMC10716100; doi:10.1007/s12195-023-00772-0)
Supplement: Supplementary file 1 — (PDF 7435 kb) [file 12195_2023_772_MOESM1_ESM.pdf]

Supplementary Figures

The effect of substrate stiffness on elastic force transmission in the epithelial monolayers over short timescales

Tervonen A, Korpela S, Nymark S, Hyttinen J & Ihalainen TO

Cellular and Molecular Bioengineering

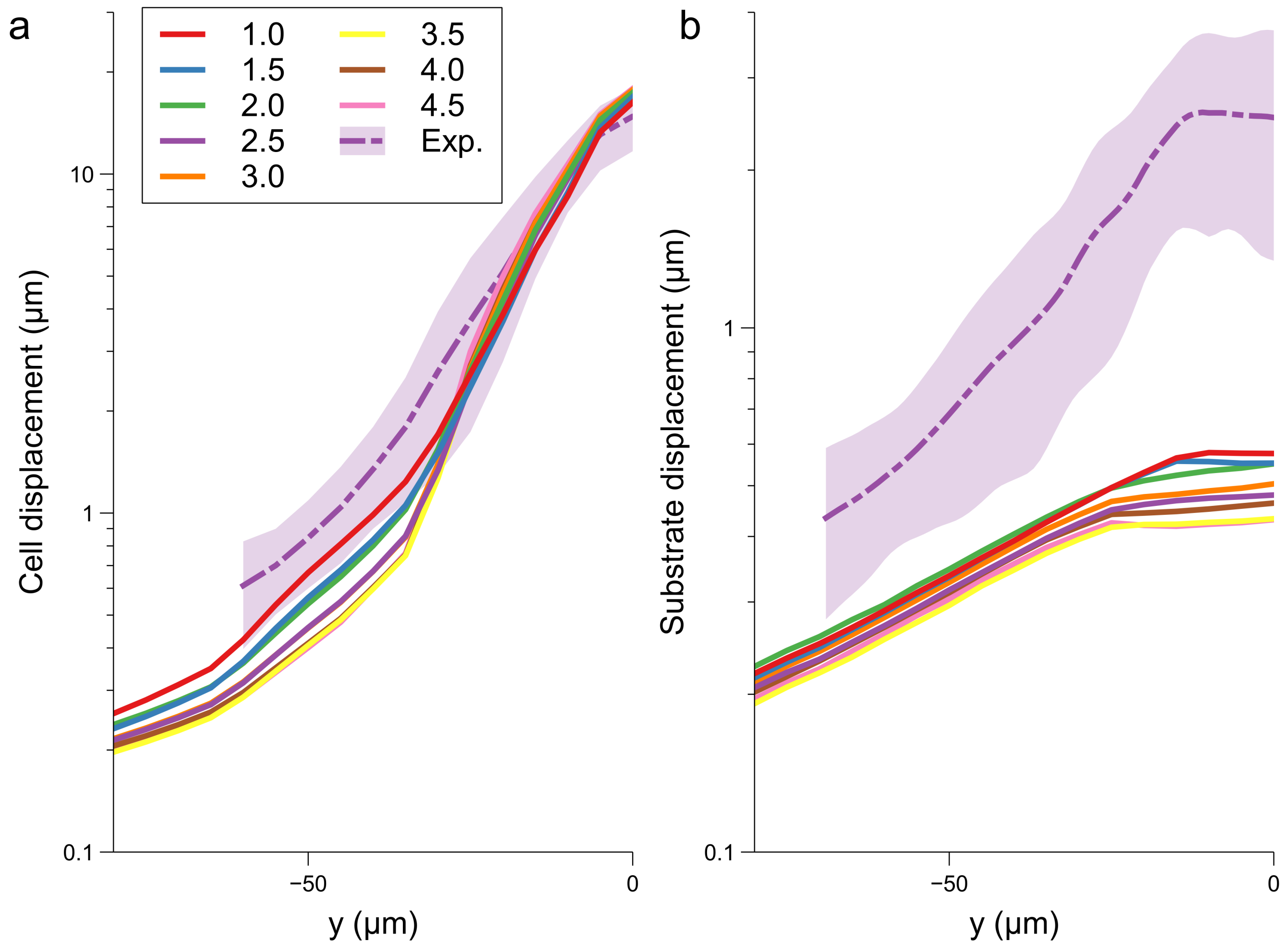

Figure S1. Simulations of epithelial on 35 kPa substrate with different values of focal adhesion strengths (unit g s^{-2} μm^{-1}) for (a) cell and (b) displacements compared to the experimental data.

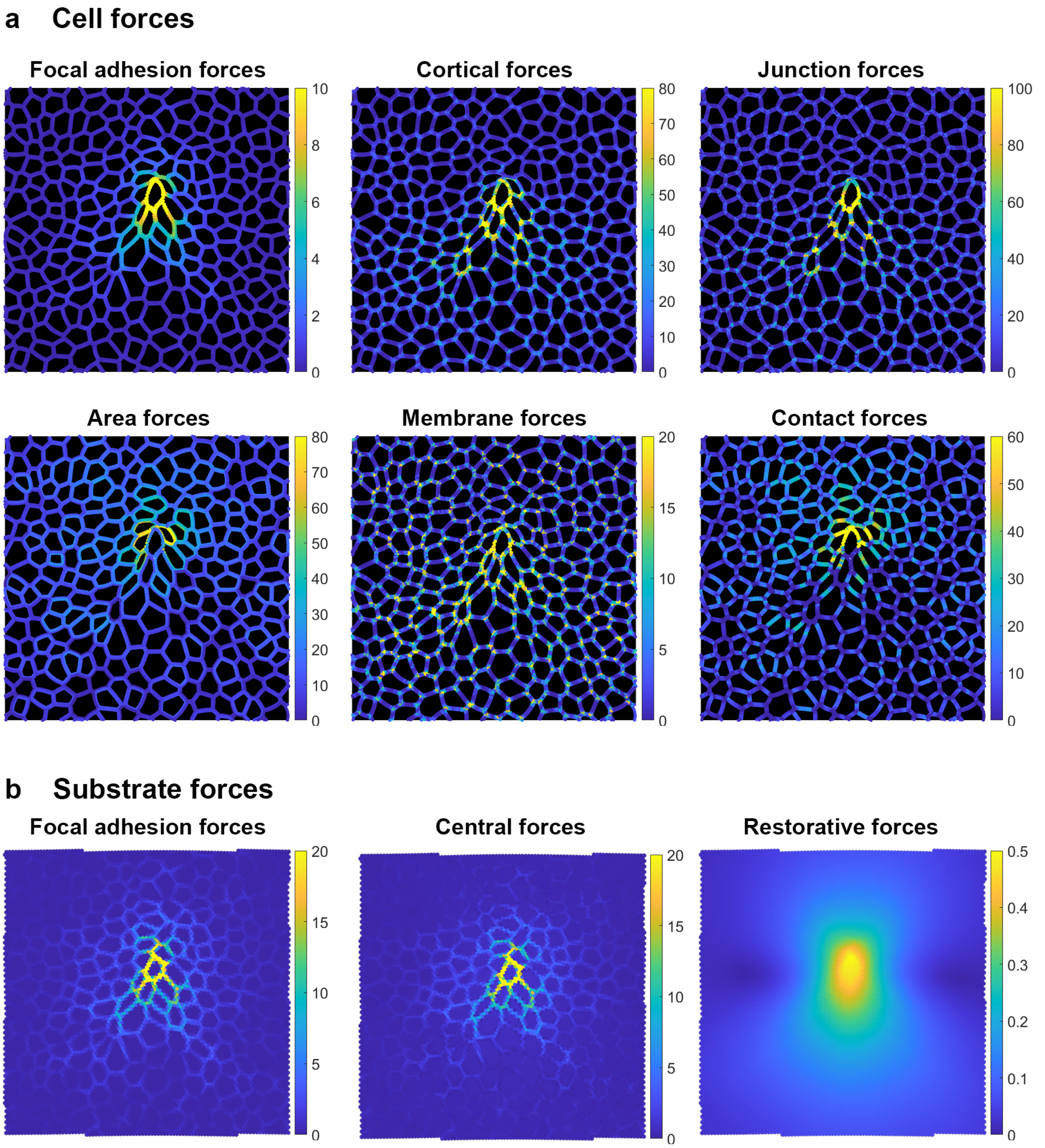

Figure S2. Representative example of cell and substrate forces following a micromanipulation. (a) Cell forces of cells on a 1.1-kPa substrate following the 30-μm micromanipulation. The values are in arbitrary units. The micromanipulation force itself is not shown as it only affected a single cell. (b) Substrate forces following the same micromanipulation. The substrate repulsion force is not shown as it was zero everywhere in the simulated area with this soft substrate.

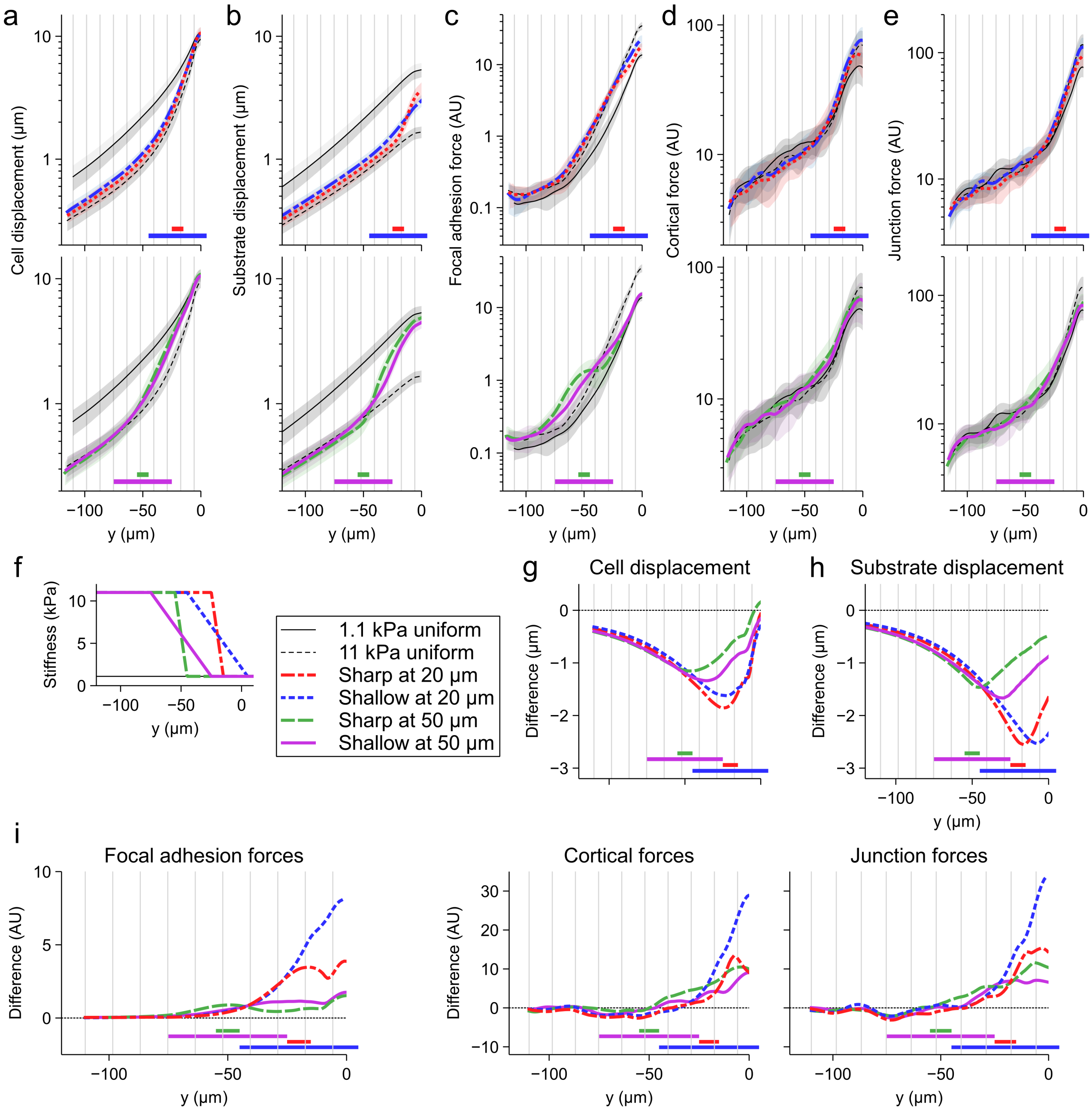
a
b
c
d
e
Cell displacement (µm)
Substrate displacement (µm)
Focal adhesion force (AU)
Cortical force (AU)
Junction force (AU)
y (µm)
f
Stiffness (kPa)
1.1 kPa uniform
11 kPa uniform
Sharp at 20 µm
Shallow at 20 µm
Sharp at 50 µm
Shallow at 50 µm
g
Cell displacement
h
Substrate displacement
Difference (µm)
i
Focal adhesion forces
Cortical forces
Junction forces
Difference (AU)

Figure S3. Vertical cell displacement and focal adhesion, cortical, and junction force propagation from soft to stiff substrate with sharp and shallow stiffness gradients. Vertical cell displacement (a), substrate displacement (b), focal adhesion forces (c), cortical forces (d), and junction forces (e) for the four different stiffness gradients shown in (f). For clarity, the four cases were divided into two figures. The corresponding plots for the uniforms stiffnesses are also shown for comparison. The forces were defined by calculating the mean force from the vertices for each cell, and then assigning the mean force for the cell centers and interpolating the values over the epithelium area. Finally, the force area data was averaged. The shaded region represents the standard deviation for each case. (f) The stiffness gradients for displacement and forces shown in (a)-(e) and (g)-(i). The absolute difference in (g) cell and (h) substrate displacement compared to the uniform 1.1-kPa displacement for stiffness gradients shown in (f). (i) The absolute differences in focal adhesion, cortical, and junction forces for stiffness gradients shown in (f) compared to the forces in the corresponding position with 1.1-kPa substrate. The vertical striping shows the positions of cell boundaries for average sized cells and the positions of the gradients are shown with the lines of corresponding colors at the bottom of each figure. For each set of parameters, n = 15. AU, arbitrary unit.

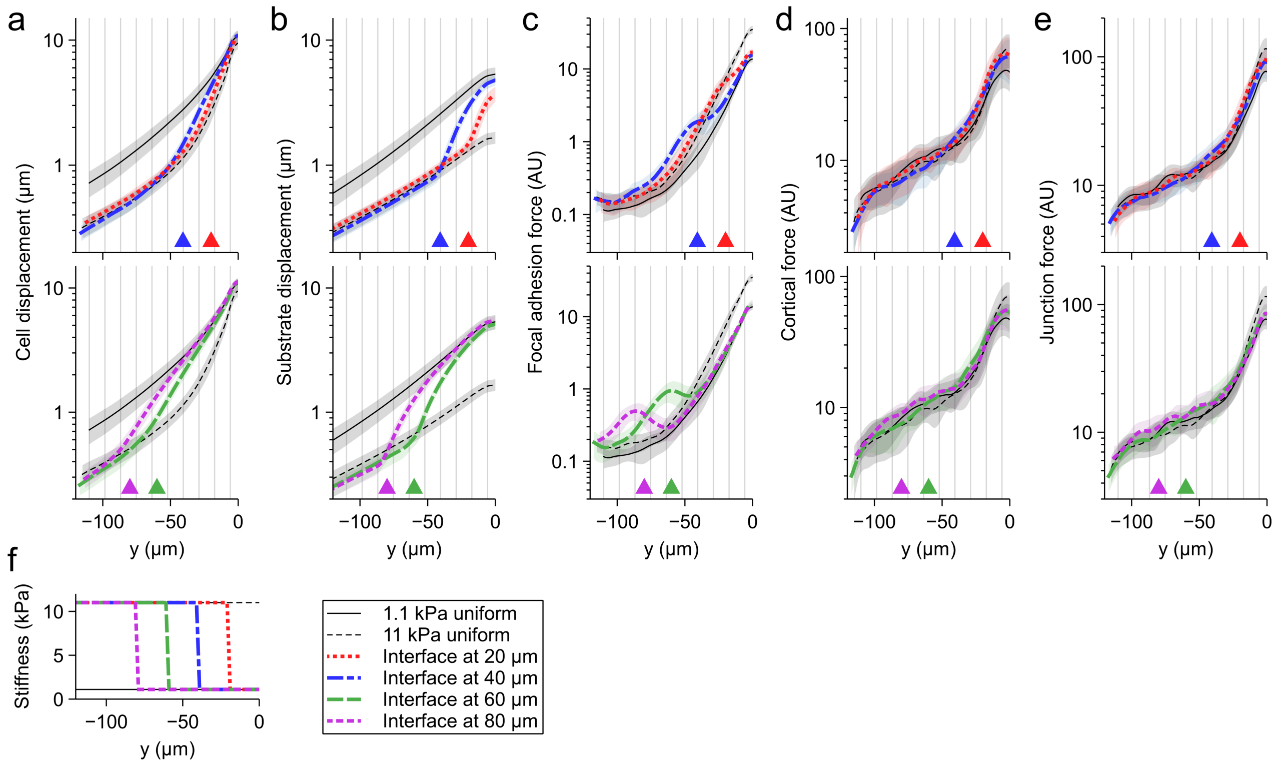

Figure S4. Vertical cell displacement and focal adhesion, cortical, and junction force propagation from soft to stiff substrate with stiffness interface gradients. Vertical cell displacement (a), substrate displacement (b), focal adhesion forces (c), cortical forces (d), and junction forces (e) for the four different stiffness interface gradients shown in (f). For clarity, the four cases were divided into two figures. The corresponding plots for the uniforms stiffnesses are also shown for comparison. The vertical striping shows the positions of cell boundaries for average sized cells and the positions of the interfaces are shown with the arrowheads of corresponding colors at the bottom of each figure. The forces were defined by calculating the mean force from the vertices for each cell, and then assigning the mean force for the cell centers and interpolating the values over the epithelium area. Finally, the force area data was averaged with n = 15 simulations. (f) The stiffness interfaces for displacement and forces shown in (a)-(e). The shaded region represents the standard deviation for each case. The force magnitudes are comparable between each other. AU, arbitrary unit.

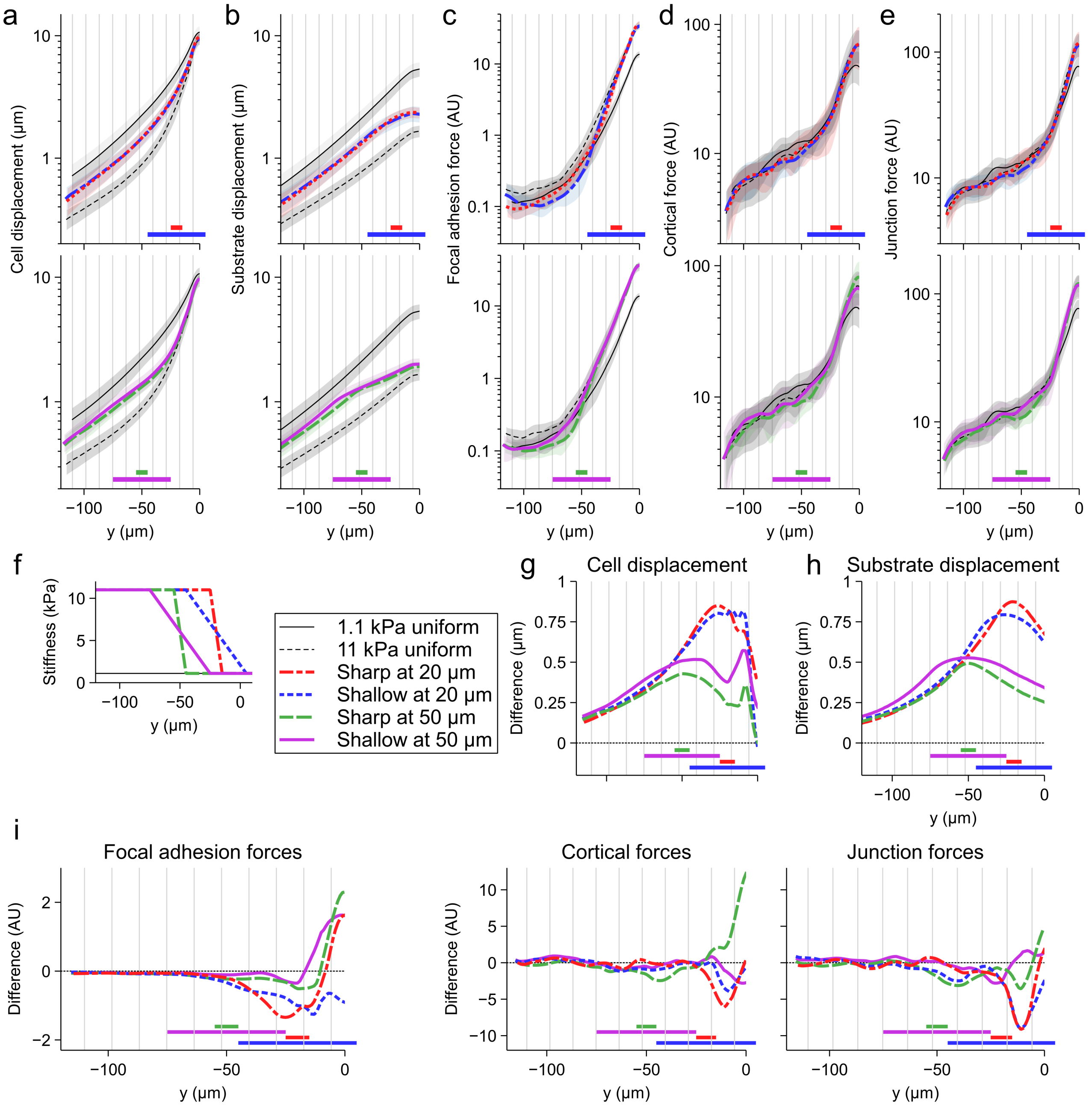
a
b
c
d
e
Cell displacement (µm)
Substrate displacement (µm)
Focal adhesion force (AU)
Cortical force (AU)
Junction force (AU)
y (µm)
f
Stiffness (kPa)
1.1 kPa uniform
11 kPa uniform
Sharp at 20 µm
Shallow at 20 µm
Sharp at 50 µm
Shallow at 50 µm
g
Cell displacement
h
Substrate displacement
Difference (µm)
i
Focal adhesion forces
Cortical forces
Junction forces
Difference (AU)

Figure S5. Vertical cell displacement and focal adhesion, cortical, and junction force propagation from stiff to soft substrate with sharp and shallow stiffness gradients. Vertical cell displacement (a), substrate displacement (b), focal adhesion forces (c), cortical forces (d), and junction forces (e) for the four different stiffness gradients shown in (f). For clarity, the four cases were divided into two figures. The corresponding plots for the uniforms stiffnesses are also shown for comparison. The forces were defined by calculating the mean force from the vertices for each cell, and then assigning the mean force for the cell centers and interpolating the values over the epithelium area. Finally, the force area data was averaged. The shaded region represents the standard deviation for each case. (f) The stiffness gradients for displacement and forces shown in (a)-(e) and (g)-(i). The absolute difference in (g) cell and (h) substrate displacement compared to the uniform 1.1-kPa displacement for stiffness gradients shown in (f). (i) The absolute differences in focal adhesion, cortical, and junction forces for stiffness gradients shown in (f) compared to the forces in the corresponding position with 1.1-kPa substrate. The vertical striping shows the positions of cell boundaries for average sized cells and the positions of the gradients are shown with the lines of corresponding colors at the bottom of each figure. For each set of parameters, n = 15. AU, arbitrary unit.

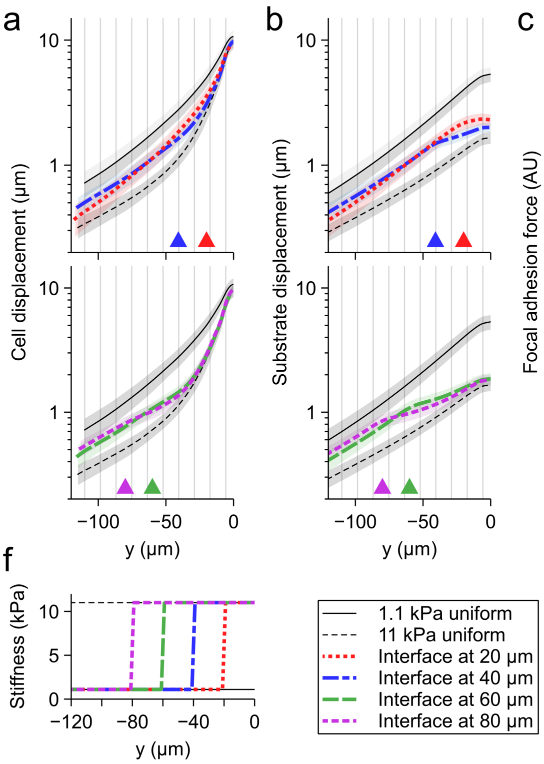

Figure S6. Vertical cell displacement and focal adhesion, cortical, and junction force propagation from stiff to soft substrate with stiffness interface gradients. Vertical cell displacement (a), substrate displacement (b) focal adhesion forces (c), cortical forces (d), and junction forces (e) for the four different stiffness interface gradients shown in (f). For clarity, the four cases were divided into two figures. The corresponding plots for the uniforms stiffnesses are also shown for comparison. The vertical striping shows the positions of cell boundaries for average sized cells and the positions of the interfaces are shown with the arrowheads of corresponding colors at the bottom of each figure. The forces were defined by calculating the mean force from the vertices for each cell, and then assigning the mean force for the cell centers and interpolating the values over the epithelium area. Finally, the force area data was averaged with n = 15 simulations. (f) The stiffness interfaces for displacement and forces shown in (a)-(e). The shaded region represents the standard deviation for each case. The force magnitudes are comparable between each other. AU, arbitrary unit.
