# Supplementary material for: The Effect of Substrate Stiffness on Elastic Force Transmission in the Epithelial Monolayers over Short Timescales
Source: Cell Mol Bioeng. 2023 Jul 13;16(5-6):475–95. doi: 10.1007/s12195-023-00772-0 (PMC10716100; doi:10.1007/s12195-023-00772-0)
Supplement: Supplementary file 2 — (PDF 3048 kb) [file 12195_2023_772_MOESM2_ESM.pdf]

Supplementary Text: Description of the model

The effect of substrate stiffness on elastic force transmission in the epithelial monolayers over short timescales

Tervonen A, Korpela S, Nymark S, Hyttinen J & Ihalainen TO

Cellular and Molecular Bioengineering

1 Model description

The model was implemented using MATLAB (R2020b, The MathWorks Inc., Natick, Massachusetts) and the model code is available at https://github.com/atervn/epimech and achieved in Zenodo [1]. In the model, the epithelium is described as a two-dimensional monolayer and thus each cell is represented by a closed polygon as its boundary. Cellular structures and processes are incorporated into the model as forces affecting the polygon vertices. The top surface of the underlying substrate is represented by a two-dimensional triangular grid of points. As with the cells, the mechanics of the substrate grid are represented by forces acting on the points. The initial epithelium for the mechanical simulations is formed by simulation epithelial growth from a single cell without the substrate. The cells are only allowed to divide during the growth simulations.

To evolve the model system during the simulation, the equation of motion is used. The system is assumed to be overdamped, enabling the omission of inertial effects. This simplification is commonly done as the importance of inertia is small in biological systems [2, 3, 4]. The movement of cell vertex i and substrate point m are calculated as

$$\eta \frac{d\vec{r}_i}{dt} = \vec{F}_{i,tot}, \tag{1}$$

$$\eta \frac{d\vec{s}_m}{dt} = \vec{F}_{m,tot}, \tag{2}$$

where η is the dampening coefficient (kg s^{-1}), $\vec{r}_i$ is the position of the cell vertex i (m), $\vec{s}_m$ is the position of the substrate point m (m), t is time (s), and $\vec{F}_{i,tot}$ is the total force acting on cell vertex i (N) and $\vec{F}_{m,tot}$ that on the substrate point m (N).

The forces included for the cell vertices include cortical actomyosin tension, cell-cell junctions, intracellular pressure, cell division, focal adhesions, membrane elasticity, contact between cells, and an edge force to describe the continuity of the epithelium outside the simulated area. The total force for each cell vertex i is therefore calculated as the sum of these component forces:

$$\vec{F}_{i,tot} = \vec{F}_{i,cort} + \vec{F}_{i,junc} + \vec{F}_{i,area} + \vec{F}_{i,div} + \vec{F}_{i,fa} + \vec{F}_{i,mem} + \vec{F}_{i,cont} + \vec{F}_{i,edge}, \tag{3}$$

where $\vec{F}_{i,cort}$ is the cortical actomyosin force (N), $\vec{F}_{i,junc}$ the cell-cell junction force (N), $\vec{F}_{i,area}$ the area force that describes the internal pressure (N), $\vec{F}_{i,div}$ the division force (N), $\vec{F}_{i,fa}$ the focal adhesion force (N), $\vec{F}_{i,mem}$ the membrane force (N), and $\vec{F}_{i,cont}$ the contact force (N).

The mechanics of the substrate are divided into three forces: a central force between neighboring points, a repulsive force between a point and the connection between two of its neighbors, and a restorative force that seeks to move a point to its original location. The second force is included to prevent the collapse of the material during large deformations [5], and the third to describe the fact that the substrate is attached to rigid glass at its bottom surface in our measurements. Furthermore, a fourth force component is included to depict the cell-substrate connection via the focal adhesions. Now, the total force affecting each substrate point is calculated as

$$\vec{F}_{m,tot} = \vec{F}_{m,cent} + \vec{F}_{m,rep} + \vec{F}_{m,rest} + \vec{F}_{m,fa}, \tag{4}$$

where $\vec{F}_{m,cent}$ is the central force between closest neighboring points (N), $\vec{F}_{m,rep}$ is the repulsive force to prevent material collapse (N), $\vec{F}_{m,rest}$ is a restorative force (N), and $\vec{F}_{m,fa}$ is the force from the focal adhesions (N).

2 Cell Components and processes

2.1 Cell membrane

The cell membrane is described by the vertices of the polygon. The main aim of the membrane force in the model is to keep the cell vertices at a given distance from each other. New cell vertices are added – e.g. during the growth – when the distance between two vertices becomes twice the normal membrane length l_{mem} (m), and vertices are removed when this distance is less than $l_{mem}/2$. In addition, to prevent narrow cell regions during the growth that might lead to the cell polygon intersecting itself, vertices with an angle less than $\theta_{mem,max}$ are removed.

2.2 Actomyosin cortex

Tension in the apical actomoysin cortex is modeled as interactions between every second cell vertex in the polygon. If a vertex is concave, the cortical link between its neighboring vertices travels around the concave vertex and pushes it outwards. The magnitude of the cortical tension forms from two components: The first component represents the normal continuous tension in the prestressed cortex, whereas the second describes the

viscoelastic behavior of the cortex by resisting rapid changes in cell perimeter. The effect of the perimeter is included in the cortical force instead of the membrane force to prevent the membrane from forming a sawtooth pattern while aiming to retain the perimeter. Now, the total magnitude of the cortical force is calculated as $(1+C_{per}\epsilon_k)k_{cort}$, where C_{per} is a perimeter-tension constant relating the changes in perimeter strain, ϵ_k, to the changes in cortical tension, and k_{cort} is a force constant describing the continuous cortical tension (kg s^{-2}).

The normal cell perimeter is remodeled as a function of the perimeter strain based on the equations derived for the vertex model by Staddon et al. [6]. The normal perimeter is described by the equation

$$\frac{dP_{k,0}}{dt} = r_{per}\epsilon_k P_{k,0} \tag{5}$$

where $P_{k,0}$ is the normal perimeter of cell k (m), r_{per} is the perimeter remodeling rate (s^{-1}), and ϵ_k is the strain in the perimeter of cell k, i.e. $\epsilon_k = (P_k - P_{k,0})/P_{k,0}$, where P_k is the current perimeter of cell k (m)[6].

2.3 Cell-cell junctions

The cell-cell junctions are described by intercellular connections between two vertices. A vertex can form a junction connection with two vertices in two separate neighboring cells. New junction connections can be thus be formed between vertices with no or with one existing junction connections given some limitations: 1) the distance between the vertices to be connected cannot exceed $2l_{junc}$, where l_{junc} is the junction rest length (m); 2) the angle of the junctions in relation to the membrane normal in each cell cannot be more than $C_{junc,max}\theta_{mem}/2$, where $C_{junc,max}$ is the maximum junction angle constant and θ_{mem} is the outside membrane angle; and 3) the new junction cannot intersect with neighboring junctions. The aim is to optimize the connections so that a vertex is linked to another as close as possible.

The junction connections are removed based on two criteria: 1) If the angle between the junction connection and the membrane normal becomes too large ($> C_{junc,max}\theta_{mem}/2$), or 2) if the distance between the connected vertices exceeds $2l_{junc}$. The first criterium is important to allow the cells to slide against each other over long timescales. Changes in the junctions, both adding and removal, are conducted only at time points defined by junction modification time step dt_{junc} (s) to reduce the computational load of these modifications.

2.4 Cell division

Cells can divide when the epithelium is grown. The division process is modeled in five phases: growth initiation, growth, division axis definition, cytokinesis, and final cleaving. The process and changes between the different phases are presented in Fig. 1a. The initiation of the growth is different for the cells on the edge and in the middle of the epithelial tissue. The cells on the edge are given a time from a normal distribution after which they initiate the growth. For a cell to become an internal cell, it must have a continuous section of vertices without any junction connections shorter than 10 % of the total number of vertices in the cell. The internal cells are also given a division time from a normal distribution but have a probability to begin the division process after this time has passed defined as $\rho_{div}\Delta t$, where ρ_{div} is the rate of division in a given time (s^{-1}) and Δt is the time step (s). Because of contact inhibition between cells when the cell density increases, the value of ρ_{div} has been shown to decrease as the cell apical area decreases [7]. The following equation was derived for the growing MDCK epithelial colony:

$$\rho_{div} = \rho_{div,0}A_k^{\alpha_{div}} \tag{6}$$

where $\rho_{div,0}$ is the basal division rate (m^{-6} s^{-1}), A_k is the current area of cell k (m^2), and α_{div} is a constant describing the division rate's dependency on the cell area [7].

Before the growth is initiated, the normal areas of the daughter cells are determined. To produce an epithelium with uniform cell size, these normal areas can be set to that of the mother cell. However, to produce an epithelium with varying cell sizes, the new normal areas of the daughter cells can be defined from a given size distribution. Both daughter cell areas must be larger than the minimum cell size A_{min} and neither can have an area more than 1.5 times that of the others. The minimum area was used to restrict the formation of small cells that sometimes led to artifacts within the epithelium by being flattened between larger cells. Also, the combined area of the daughter cells must be between 1.5 and 2.5 times that of the mother cell. These restrictions are used to prevent too fast changes in cell sizes during growth. Furthermore, since cortical tension by default tries to reduce the cell size, the daughter cell areas are multiplied by constant C_{area} to compensate for area reduction of the cortical tension.

The cell growth is accomplished by using the area force. Therefore, to begin the cell growth, the normal area of the cell is set to $C_{growth}(A_{k,1} + A_{k,2})$, where C_{growth} is a constant used to control the cell growth rate, and $A_{k,1}$ and $A_{k,2}$ are the areas of the two daughter cells of cell k (m^2). While the cell grows, new vertices are added to the membrane.

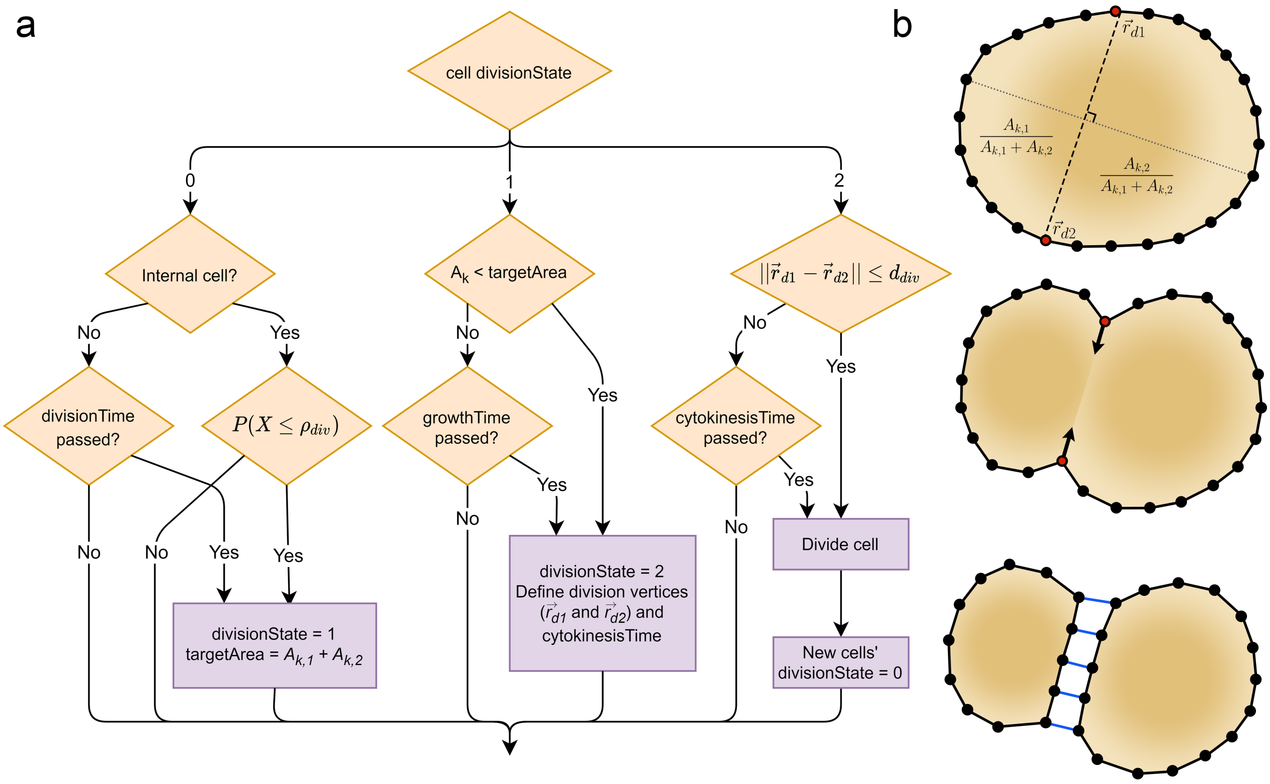

Figure 1: (a) The cell division phases presented in a flow chart for a single time step of the simulation. The cells can have three different division states: 0, quiescent state; 1, growing; and 2; dividing. The way that cells in the quiescent state progress depend if they are outer or internal cells. The outer cells wait until a predefined division time after which they automatically change to the growing state. To include contact inhibition to the internal cells, their probability to begin the growing process depends on a probability defined by Eq.6. Cells that transfer from the quiescent to the growing state are assigned areas for the future daughter cells ($A_{k,1}$ and $A_{k,2}$), whose combined area is set to be the target to which the cell has to grow to in order to begin division. The cell in the growing state increases in apical area A_k until is reaches the target area, at which state division vertices ($\vec{r}_{d1}$ and $\vec{r}_{d2}$) are defined based on the daughter cell areas and Hertwig's rule. If the cells cannot reach the target area within a defined growth time limit, they will be transferred to the dividing state. In the dividing state, the division vertices are moved towards each other until the are at a distance equal or smaller than a predefined division distance, in which case they divide to form two new cells whose division state is set to 0. If for some reason, the division vertices cannot be brought to the division distance, the cell will undergo cytokines after a defined time limit. (b) The phases of the division. Top: the definition of the division vertices based on the dashed line that is perpendicular to the longest cells axis (dotted line) based on Hertwig's rule, and that divides the cells according to the relative areas of the daughter cells. Middle: The movement of the division vertices towards each other by a division force. Bottom: The formation of the daughter cells after either the division vertices reach the limit distance or the cytokinesis time limit is passed.

The growth ends in two ways: 1) the cell area surpasses the area of $A_{k,1} + A_{k,2}$ or 2) the defined maximum growth time $t_{max,growth}$ (s) has passed from the beginning of the growth. The latter case prevents the situations in which cell growth is inhibited by the surrounding cells. Once the growth has ended, the division axis is defined. This is done based on Hertwig's rule, where the division axis is set perpendicular to the longest axis of the cell (see Fig. 1b) [8]. The location of the division axis along the longest axis is set so that it divides the mother cell area in the ratio between the daughter cell areas. Vertices on both sides of the cell closest to this axis are appointed as the division vertices.

Next, the division vertices are moved towards each other by the division force as shown in Fig. 1b. Once the distance between the vertices is smaller than a specified division distance or a maximum cytokinesis time $t_{max,div}$ (s) has passed, the cell will divide. The division distance is calculated as division distance constant C_{div} times the initial distance between the division vertices. The division is done by connecting the vertices on each side of the division vertices to form the new sections of membrane for the daughter cells (see Fig. 1b). Then, new vertices are inserted into these new sections to obtain the desired vertex separation.

2.5 Focal adhesions

To describe the focal adhesions between the cells and the underlying substrate, each cell vertex is attached directly to the substrate at its original position. In practice, each attachment position is within a triangle formed by three closest substrate points. The coordinates of the attachment position are calculated as a weighted average of those of the three closest substrate points. A barycentric coordinate system is used to define these weights so that the deformation of the substrate does not affect the relative focal adhesion position. The weights for each focal adhesion can be solved from the equations

$$\begin{cases} x_{i,a} = \sum_{m=1,2,3} w_{i,m} x_m \\ y_{i,a} = \sum_{m=1,2,3} w_{i,m} y_m \\ w_{i,1} + w_{i,2} + w_{i,3} = 1 \end{cases} \tag{7}$$

where $x_{i,a}$ and $y_{i,a}$ are the coordinates of the attachment position of cell vertex i, x_m and y_m are the coordinates of the three closest substrate points, and $w_{i,m}$ $(m = 1, 2, 3)$ are the weights for those coordinates. In addition, the focal adhesions can be broken individually for each vertex when the focal adhesion force per membrane length is over $F_{fa,max}$ (N μm^{-1}). When new vertices are added to the cell membrane, the new vertex will have a focal adhesion if both the neighboring vertices have intact focal adhesions. This new focal adhesion will be formed with the substrate position halfway between the positions of those of the neighboring vertices.

3 Substrate stiffness

In the simulation with the substrate, the substrate stiffness can be described in various ways. In the simplest case, the substrate stiffness is uniform, with every substrate point having the same properties. Stiffness gradients are produced by defining the stiffness as a function of location along a chosen direction. This method can also be used to create sharp interfaces in stiffness.

The substrate stiffness can also be defined based on a random heterogeneous profile. The size of the features in the profile depends on the surface autocorrelation lengths defined separately for x- and y-directions. In addition, the profile can be rotated to obtain autocorrelation behavior in any direction.

4 Force components

In the equation, the following notation is used: $\vec{r}_i = (x_i, y_i)$, the coordinates of cell vertex i; $\vec{s}_m = (x_m, y_m)$, the coordinates of substrate point m; $\vec{d}_{i,j} = \vec{r}_j - \vec{r}_i$ vector between two cells vertices or substrate points i and j; $d_{i,j} = \|\vec{d}_{i,j}\|$, length of the vector between two cell vertices or substrate points i and j; and $\hat{d}_{i,j} = \vec{d}_{i,j}/\|\vec{d}_{i,j}\|$, the unit vector in the direction of vector $\vec{d}_{i,j}$.

4.1 Cortical force

The actomyosin cortex is described by interactions between every other vertex in the cell membrane, i.e. vertex i is connected with the vertices $i+2$ and $i-2$. If the nearest neighboring vertex on either side is concave, the force is directed towards this nearest vertex. Since a cortical connection travels around a concave vertex on its intracellular side, a force pushing the concave vertex outwards is included. The cortical forces are calculated as

$$\vec{F}_{i,cort} = (1 - C_{per}\epsilon_k)k_{cort} \times \begin{cases} \left(b_{i,i-2}d_{i,i-2}\widehat{d}_{i,n_-} + b_{i,i+2}d_{i,i+2}\widehat{d}_{i,n_+}\right) & \text{, if } i \text{ is convex} \\ \left(b_{i,i-2}d_{i,i-2}\widehat{d}_{i,n_-} + b_{i,i+2}d_{i,i-2}\widehat{d}_{i,n_+}\right) - b_{i-1,i+1}d_{i-1,i+1}\left(\widehat{d}_{i-1,i} + \widehat{d}_{i+1,i}\right) & \text{, if } i \text{ is concave} \end{cases} \tag{8}$$

where the force magnitude part was described in subchapter 2.2. The constants $b_{i,j}$ describe the individual stiffness of the cortical interaction between vertices i and j and can be used to change the local stiffness of the cortex. The indices n_- and n_+ depend on whether the previous or next vertices, respectively, are convex or concave. For convex neighbors, the values are $n_- = i - 2$ and $n_+ = i + 2$, and for concave ones, $n_- = i - 1$ and $n_+ = i+1$. The second factor for the concave vertices describes the additional force acting on the concave vertex and is calculated as the negative of the sum of the forces acting on the neighboring vertices by the cortical link that connects them.

4.2 Junction force

The junction force affecting two intercellular vertices i and j is described by a linear spring as follows

$$\vec{F}_{i,junc} = k_{junc}\left(d_{i,j} - l_{junc}\right)\widehat{d}_{i,j} \tag{9}$$

where k_{junc} is the junction force coefficient (kg s^{-2}), and l_{junc} is the junction rest length (m).

4.3 Area force

Area force is used to describe the effect of intracellular pressure and the tendency of a cell to preserve its apical area. The area force for a vertex i in cell k is calculated similarly to boundary-based models by Tamulonis et al.[9] and the vertex models assuming that the force is directly proportional to the relative difference between the current area and the normal area[2, 3]

$$\vec{F}_{i,area} = -k_{area}\left(\frac{A_k - A_{k,0}}{A_{k,0}}\right)\begin{bmatrix} y_{i+1} - y_{i-1} \\ x_{i-1} - x_{i+1} \end{bmatrix} \tag{10}$$

where k_{area} is the cell area elastic modulus (kg s^{-2}), A_k is the current apical area of cell k (m^2), and $A_{k,0}$ is the normal apical area of cell k (m^2). The relative change in area is calculated instead of the absolute change to make k_{area} independent of the normal area. To control the rate of the cell growth, the area force is multiplied by an additional constant $C_{area,growth}$ during the growth phase.

4.4 Division force

Division force between the two defined division vertices is used to model cytokinesis during cell division. A force between division vertices i and j is given as

$$\vec{F}_{i,div} = k_{div}\widehat{d}_{i,j} \tag{11}$$

where k_{div} is the division force constant (N).

4.5 Focal adhesion force

Focal adhesions are described by connections between a cell vertex and position in the substrate that is defined as a weighted average of the three closest substrate points. The force exerted on a cell vertex i depends on the coordinates of the three initially closest substrate points ($m = 1, 2, 3$) and their force is calculated based on the weights for each substrate point as follows

$$\vec{F}_{i,fa} = \begin{bmatrix} \sum_{m=1,2,3} k_{fa}\left(E_m\right) l_{mem}\left(\left(w_{i,m}x_m\right) - x_i\right) \\ \sum_{m=1,2,3} k_{fa}\left(E_m\right) l_{mem}\left(\left(w_{i,m}y_m\right) - y_i\right) \end{bmatrix}, \tag{12}$$

where $k_{fa}\left(E_m\right)$ is the focal adhesion force strength per unit length (kg s^{-2} μm^{-1}) that depends on Young's modulus E_m of the substrate point m (Pa), and $w_{i,m}$ are the substrate point weights as explained in subchapter 2.5.

The force exerted on a substrate point can depend on multiple cell vertices. Thus, this force is a sum of all those interactions and is calculated as

$$\vec{F}_{m,fa} = -\sum_{i=1}^{n} w_{i,m}\vec{F}_{i,fa}, \tag{13}$$

where n is the number of focal adhesions that depend on the position of substrate point m.

4.6 Membrane force

The membrane force is used to retain a certain distance between the cell vertices. This force is based on the nonlinear spring equation defined by Cooper & Maddock[5] for each vertex i as

$$\vec{F}_{i,mem} = \sum_{j=i-1,i+1} k_{mem} \begin{cases} \left(d_{i,j} - \frac{{l_{mem}}^2}{d_{i,j}}\right)\widehat{d}_{i,j}, & \text{if } d_{i,j} \leq l_{mem} \\ \left(d_{i,j} - \frac{{l_{mem}}^2}{d_{i,j}-2l_{mem}} - 2l_{mem}\right)\widehat{d}_{i,j}, & \text{if } d_{i,j} > l_{mem} \end{cases}, \tag{14}$$

where k_{mem} is the membrane force constant (kg s^{-2}). The part with $d_{i,j} < l_{mem}$ was derived by Cooper & Maddock, and the other component was derived to prevent the spring extension above $2l_{mem}$ with a similar formulation.

4.7 Contact force

Following the work by Tamulonis et al.[9], a force component is included so that the cells repel each other to prevent overlap. Only the interactions between vertex i and the membrane sections on either side of the closest vertices in two different neighboring cells are considered. However, these vertices have to be within a given maximum distance $l_{cont,limit}$ (m). To determine how vertex i interacts with the other cells, it is projected on the two lines running through the closest vertex j and its neighbor $j-1$ as well as j and $j+1$. A normalized projection between the vertices defining the line can be calculated as

$$u_{i,j,k} = \frac{(\vec{r}_i - \vec{r}_j)\vec{e}_{j,k}}{\vec{e}_{j,k} \cdot \vec{e}_{j,k}}, \tag{15}$$

where $e_{j,k}$ is the vector between vertices j and k, where k is either $j-1$ or $j+1$.

The location of this projection in relation to the vertices defining the line (j and $j+1$ or j and $j-1$) determine three different types of interactions:

1. The projections on both lines are between the vertices defining the lines ($u_{i,j,j-1} \in (0,1)$ and $u_{i,j,j+1} \in (0,1)$)
2. The projection on one line is between the vertices defining the lines ($u_{i,j,j-1} \in (0,1)$ and $u_{i,j,j+1} \notin (0,1)$ or $u_{i,j,j-1} \notin (0,1)$ and $u_{i,j,j+1} \in (0,1)$)
3. The projections on both lines are not between the vertices defining the lines $u_{i,j,j-1} \notin (0,1)$ and $u_{i,j,j+1} \notin (0,1)$)

The contact force is nonzero only when vertex i is closer than the junction rest length l_{junc} to the surfaces of the closest cells. Therefore, the contact force for the interaction is calculated with equation

$$\vec{F}_{cont}(\vec{r}_i, \vec{r}_k) = \begin{cases} k_{cont}\left(d_{k,i} - \frac{{l_{junc}}^2}{d_{k,i}}\right)\widehat{d}_{k,i}, & \text{if } d_{k,i} \leq l_{junc} \\ 0, & \text{otherwise} \end{cases}, \tag{16}$$

where r_k is the closest position on the surface of the neighboring cell to vertex i. Based on the three different types of interactions stated above, the resulting contact force from a nearby cell is calculated as

$$\vec{F}_{cont} = \begin{cases} \vec{F}_{cont}(\vec{r}_i, \vec{p}_{i,j,j-1}) + \vec{F}_{cont}(\vec{r}_i, \vec{p}_{i,j,j+1}), & \text{if } u_{i,j,j-1} \in (0,1) \text{ and } u_{i,j,j+1} \in (0,1) \\ \vec{F}_{cont}(\vec{r}_i, \vec{p}_{i,j,j-1}), & \text{if } u_{i,j,j-1} \in (0,1) \text{ and } u_{i,j,j+1} \notin (0,1) \\ \vec{F}_{cont}(\vec{r}_i, \vec{p}_{i,j,j+1}), & \text{if } u_{i,j,j-1} \notin (0,1) \text{ and } u_{i,j,j+1} \in (0,1) \\ \vec{F}_{cont}(\vec{r}_i, \vec{r}_j) & \text{, if } u_{i,j,j-1} \notin (0,1) \text{ and } u_{i,j,j+1} \notin (0,1) \end{cases}, \tag{17}$$

where point $p_{i,j,j\pm1}$ is the projection of vertex i on the edge between vertices j and $j \pm 1$. The vector between the point $p_{i,j,j\pm1}$ and vertex i is calculated with equation [9]

$$\vec{d}_{p,i} = \hat{e}_{j,j\pm1} \times (\vec{r}_i - \vec{r}_j) \times \hat{e}_{j,j\pm1}. \tag{18}$$

Since a vertex can have a contact force with two neighboring cells, the final contact force in these cases is the sum of these two separate contact interactions.

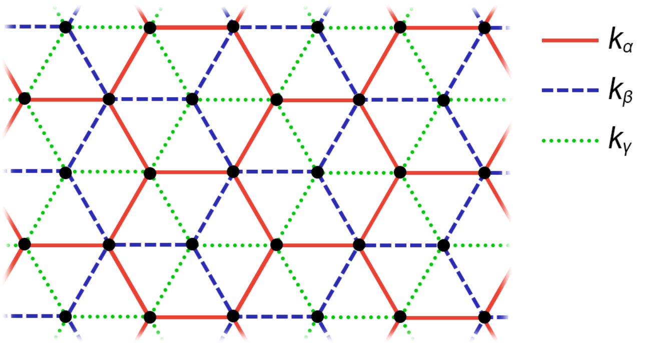

Figure 2: The triple honeycomb lattice model in which three hexagonal lattices with different spring constants are used the define the mechanics of the triangular lattice in order to obtain higher Poisson's ratios than 1/3. Figure based on refs. [12, 11]

4.8 Cell edge force

Since the epithelium continues outside the simulated area, the effect of the tissue outside the model must be considered. This is done by a force, that aims to restore the outside, non-junctional vertices of the edge cells to their original location. The force for vertex i is calculated with equation

$$\vec{F}_{i,edge} = k_{edge} l_{mem} \left(\vec{r}_{i,0} - \vec{r}_i\right) \tag{19}$$

where k_{edge} is the edge force constant per μm of the membrane (kg s^{-2} μm^{-1}), l_{mem} is the normal membrane length (m), and $r_{i,0}$ is the initial location of vertex i.

4.9 Central substrate force

The central substrate force represents the interactions between substrate point m and its direct neighbors. A material described by a triangular meshwork with only central forces always has Poisson's ratio of 1/3 [10]. To be able to modify the Poisson's ratio of the substrate, we implement the so-called triple honeycomb lattice model, in which the triangular mesh is described by three overlapping hexagonal lattices with spring constants k_α, k_β, and k_γ (Fig. 2) [11, 12]. In essence, any Poisson's ratio (ν) can be achieved by varying the relative values of these spring constants and it can be calculated with the equation[11]

$$\nu = 1 - \frac{2}{1 + \frac{2}{9}\left(k_\alpha + k_\beta + k_\gamma\right)\left(\frac{1}{k_\alpha} + \frac{1}{k_\beta} + \frac{1}{k_\gamma}\right)} \tag{20}$$

The values of k_α, k_β, and k_γ can be simplified by defining their value as relative to a single spring constant, or $k_\alpha = \alpha k_{sub}$, $k_\beta = \beta k_{sub}$, and $k_\gamma = \gamma k_{sub}$.

Now, the central substrate interactions are represented by nonlinear springs and the force can be calculated as[5]

$$\vec{F}_{m,cent} = \sum_n C_{cent} k_{sub}(E_m, E_n)\left(d_{n,m} - \frac{l_{sub}^2}{d_{n,m}}\right)\widehat{d}_{n,m}, \tag{21}$$

where C_{cent} is the constant α, β, or γ depending on the spring, k_{sub} is the central substrate spring constant (kg s^{-2}), l_{sub} is the substrate rest length, and n goes through the neighbors of point m.

The value of k_{sub} depends on Young's moduli at the substrate point m and the neighboring point n. If they have the same moduli, $k_{sub} = C_E E_m$, where C_E is a constant to relate the k_{sub} to the Young's modulus at point m (kg s^{-2} Pa^{-1}). However, if the points have different moduli, k_{sub} is calculated as[12]

$$k_{sub}(E_m, E_n) = \left(\frac{1}{2C_E E_m} + \frac{1}{2C_E E_n}\right)^{-1}. \tag{22}$$

4.10 Repulsive substrate force

The repulsive substrate force is used to prevent the substrate from collapsing by restricting the substrate point within the space limited by its direct neighbors. The force is calculated similarly to the cell contact force by calculating the force between a point and the section between two of its neighbors. However, to make the calculations faster, it is assumed that the projection of point m is always on the section between two of its

neighbors. If this is not the case, the repulsion between point m and other sections formed by its neighbors restricts its movement. The force is calculated as:

$$\vec{F}_{m,rep} = \begin{cases} \sum_n k_{sub}(E_m) \left(d_{p,m} - \frac{l_{rep}^2}{d_{p,m}} \right) \widehat{d}_{p,m}, & \text{if } d_{p,m} < l_{rep} \\ 0, & \text{otherwise} \end{cases}, \tag{23}$$

where $d_{p,m}$ is the distance between substrate point m and its projection point p on the line between two of its neighboring points as defined as with the cell contact force, l_{rep} is the distance at which the repulsion begins to take effect (m), and n goes through the edges between by the neighboring points. The repulsion distance is calculated from the substrate rest length by $l_{rep} = C_{rep} l_{sub}$, where C_{rep} is a repulsion length constant. Here, k_{sub} depends only on Young's modulus of point m.

4.11 Restorative substrate force

To describe the fact that the substrate is attached to the stiff glass at its bottom surface in our measurements, a restorative force is used to describe the substrate's desire to return to its original form. To simplify the model, we assume that the force constant for this force is directly proportional to k_{sub}. In addition, a multiplier constant is included for the points at the edge of the substrate to describe that the substrate, in reality, continues outside the simulated area. Now, the restorative force is calculated as

$$\vec{F}_{m,rest} = \begin{cases} C_{edge} k_{rest} \left(\vec{s}_{m,0} - \vec{s}_m \right), & \text{if } m \text{ is an edge point} \\ k_{rest} \left(\vec{s}_{m,0} - \vec{s}_m \right), & \text{otherwise} \end{cases}, \tag{24}$$

where k_{rest} is the restorative force constant, C_{edge} is the edge point multiplier, and $\vec{s}_{m,0}$ is the original position of substrate point s_m. The restorative force constant is calculated as

$$k_{rest} = C_{rest} A_{sub} k_{sub}, \tag{25}$$

where C_{rest} is a proportionality constant between k_{sub} and k_{rest} that depends on the hexagonal area around a substrate point, A_{sub}. A_{sub} is calculated as $A_{sub} = 2\sqrt{3}(l_{sub}/2)^2$. Again, k_{sub} depends only on Young's modulus of point m.

5 Model evolution

The model is solved using either 2nd or 4th order Runge-Kutta methods. When the epithelium is grown from a single cell and the substrate is not included in the model, 2nd order Runge-Kutta was used, since it was found to be sufficiently accurate. These simulations also omit the focal adhesion forces and the cell edge force. During simulations that include the substrate, 4th order Runge-Kutta was used to solve the system evolution.

The time step can change during the simulation to improve the system stability. We found that the substrate requires smaller time steps than the cells to be stable, and therefore, to reduce the computational time, we separated the solution of the cells and the substrate. This enabled the cells to be first solved with a larger time step followed by the substrate which was solved with multiple smaller intermediate time steps, if needed, to reach the same time point as the cells. The time stepping was made dynamic by setting maximum limits for the cell vertex or substrate point movements ($d_{c,max}$ and $d_{s,max}$, respectively). While solving either the cells or the substrate, the solution is repeated with halved time step if the movement of any vertex or point is over these limits. In addition, after solving either the cell or substrate movement, the respective time step is doubled for the next iteration if the current time step is lower than a defined maximum time step and the maximum movement of any cell vertex or substrate point is lower than a given limit ($d_{c,low}$ and $d_{s,low}$, respectively). This is presented in Fig. 3.

6 Simulations

6.1 Growth

The epithelia used in the simulations with the substrate were first grown without the substrate to reduce the computational time. The growth initiated from a single cell and the randomness in the resulting epithelium was produced by the randomness in the times between divisions and in the areas of the new cells. The new cell areas were taken from a probability density function defined based on our experimental data (described in subchapter 7.4). The area of the first cell was set as the median of the cell area data. Following the cell growth phase, the epithelium was given time to relax using the damping coefficient used in the specific simulation with

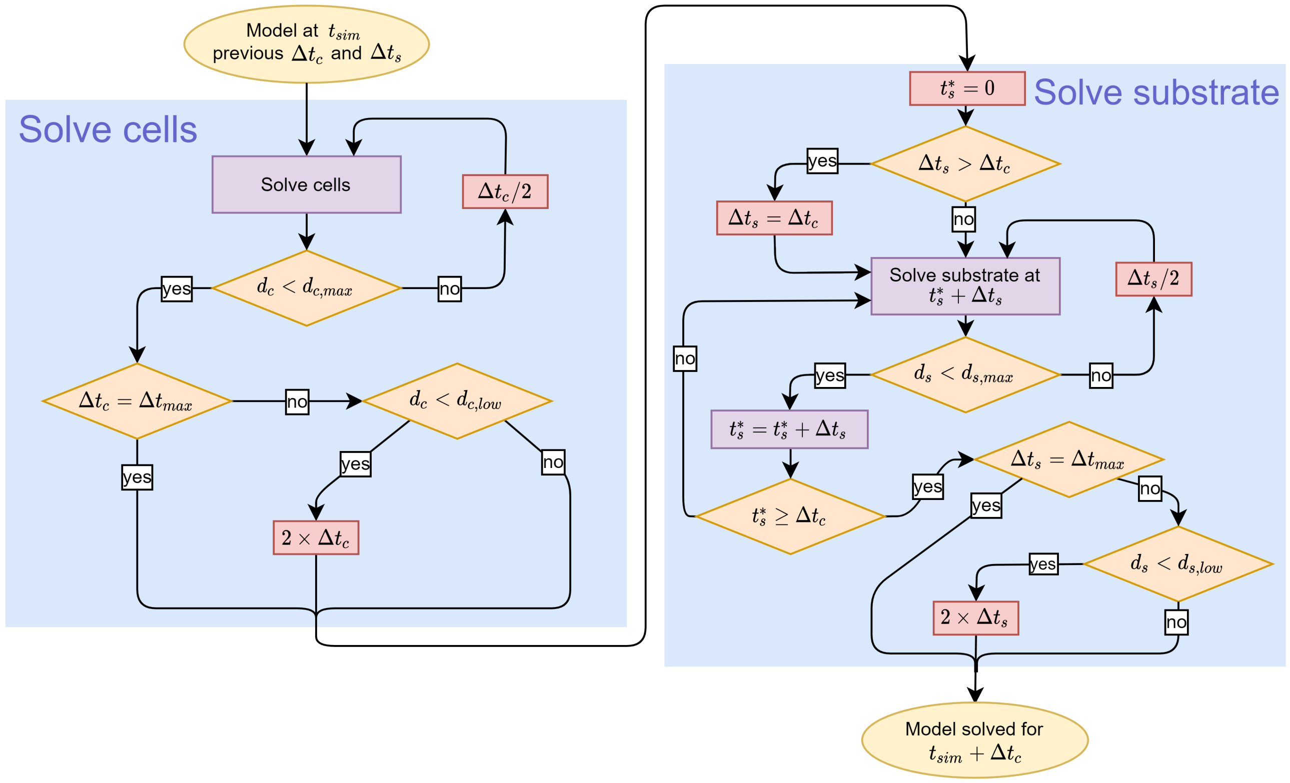

Figure 3: Description of the time stepping algorithm used in the simulations. When both the cells and the substrate are included in the model, they are solved separately to make the solution of the whole model more efficient. When only the cells are included in the growth simulations, the substrate solution phase is skipped. The solution begins by solving the movements of the cell vertices (d_c) over the cell time step (Δt_c) according to the total forces using Eq.1. Maximum vertex movement limit ($d_{c,max}$) is used to make the model stable, and if a vertex in the solution moves more that this limit, the cell movements are solved again with a halved time step. This limit check is done after solving each cell to minimize any extra calculations. When the solution contains no large vertex movements, the solution is accepted. Next, the algorithm checks if the cell time step equals the maximum time step (Δt_{max}). If no, and the maximum cell vertex movement is below a defined low movement limit ($d_{c,low}$), the cell time step is doubled for the next iteration. The low movement limit is used to prevent excess changes in the time stepping that would lead to cell movements being solved multiple times in vain. Next, the movement of the substrate vertices is solved if the substrate is included in the simulation. Since solving the substrate requires smaller time steps than the cells, a temporary substrate time (t_s^*) is defined to track the time during the substrate solution. First, the algorithm checks that the current substrate time step (Δt_s) is below the current cell time step. If not, the substrate time step is made equal to that of the cells. Next, the movement of the substrate points is solved at $t_s^* + \Delta t_s$, after which the maximum point movement is compared to the substrate movement limit ($d_{s,max}$). If too large movements are found, the substrate is solved again with halved time step. Otherwise, the temporary time is increased by After the substrate passed the movement limit check, the temporary time is increased by Δt_s. Next, the temporary time is compared to the current cell time step. If it has a value below time cell time step, the the substrate is solved again until the temporary substrate time reached the cell time step. After this, the current substrate time step is compared with the maximum time step. If the value is lower, it is doubled if the maximum substrate point movement was lower than a defined limit ($d_{s,low}$). Now, the simulation time (t_{sim}) can be progressed by the cell time step. In the next iteration, the time steps obtained from this iteration are used as the starting values.

the substrate. Then, at the beginning of the simulations with a substrate, the cell vertices are connected to it by defining the focal adhesion connections.

The size of the epithelium grown depends on the simulation. The aim is that the chosen size is suitable to show the distance of the force transduction or behavior required by each of the simulations with the substrate. The additional edge forces for the cells as well as the increased restorative force for the substrate edge points can still describe the continuity of the epithelium and thus the resistance against movement at the edges even if the simulated area itself is smaller than the distance of the force propagation.

6.2 Micromanipulation

In the micromanipulation simulations, we assumed that the short time scale of the manipulation means that the cells behave mostly elastically, and thus we ignored many of the processes in the model that relax stresses in the epithelium. Therefore, we restricted the model by prohibiting the addition or removal of cell vertices and the removal of cell-cell junctions.

To describe the micromanipulation, a cell is chosen to be moved a given distance with a known speed. This was done by including an additional micromanipulation force for this cell. We observed in the experiments that the manipulated cell did not move the same distance as the micromanipulator and that especially with higher substrate stiffnesses the segmentation of the cells near the pipette was difficult. Therefore, in the model, we decided to describe the movement of this cell by concentrating on the deformation of the surrounding cells rather than aiming to describe what happens to this single cell.

We described the movement of the micromanipulator by creating a virtual copy of the manipulated cell that was then moved the full range of the micromanipulator movement. The vertices of the real cell were connected to corresponding vertices of the virtual cell with spring. In addition, since the manipulated cell stretches in the direction of the movement, a constant depending on the positions of the vertices in relation to the movement direction was added. This constant was 1 for the vertex that was the most towards the direction of the movement and 0 for the vertex furthest away. The values for the other vertices were defined linearly between these two depending on their location. These constants were only included with the force component in the direction of the movement. Now, the force used to move the cell can be written as

$$\vec{F}_{i,mm} = \begin{bmatrix} k_{mm}\left(x_{i,mm} - x_i\right) \\ C_{mm}\left(y_i\right) k_{mm}\left(y_{i,mm} - y_i\right) \end{bmatrix}, \tag{26}$$

where k_{mm} is the force constant for the micromanipulation (kg s^{-2}), $x_{i,mm}$ and $y_{i,mm}$ are the coordinates of the virtual cell vertices that are moved (m), and C_{mm} is the position-dependent constant. The micromanipulator movement is in the direction of the y-axis.

6.3 Optogenetic activation

In the optogenetic activation simulations, the contractility of the cortex in a specific area was increased to describe the experimental activation of myosin [6]. In these simulations, a region of the epithelium was chosen for the activation. During the simulation, the activation was described by increasing the values of the constant $b_{i,j}$ for the cortical connection between vertices i and j (see Eq.8) that is at least partly within the activation region. The optogenetic activation was defined as function of time having square pulses. The magnitude of these pulses was defined as $C_{act,max} f_{act}(t)$, where $C_{act,max}$ is the maximum activation constant and f_{act} is the activation function that can have values between 0 and 1. To avoid sharp differences in cortical tension in the cells, the effect of the activation on the values of the constants $b_{i,j}$ depended on the how much of the cortical connection between i and j was within the activation region. Since the connections are between every other vertex, the value for $b_{i,i+2}$ can be

$$b_{i,i+2} = \begin{cases} 1 + C_{act,max} f_{act}(t), & \text{if } i,\ i{+}1, \text{ and } i{+}2 \text{ are within } A_{act} \\ 1 + 0.5 C_{act,max} f_{act}(t), & \text{if only } i \text{ and } i{+}1 \text{ or } i{+}1 \text{ and } i{+}2 \text{ are within } A_{act} \\ 1 + 0.25 C_{act,max} f_{act}(t), & \text{if only } i, \text{ or } i{+}2 \text{ are within } A_{act} \\ 1, & \text{if } i,\ i{+}1, \text{ and } i{+}2 \text{ are outside } A_{act} \end{cases}, \tag{27}$$

where A_{act} refers to the activation region.

7 Model parameters and fitting

7.1 Cell parameters

The cell parameters used in our simulations are presented in Table 1. We selected the value for the normal membrane length used to discretize the cell membrane (l_{mem}) to give a good resolution of the cell shape while

not being computationally too expensive. The junction rest length (l_{junc}) value was set to be in a similar size as l_{mem} to keep the cell vertices at similar distances both within the same cell and between neighboring cells. The values of the cell area elasticity (k_{area}) and the continuous cortical tension (k_{cort}) were chosen based on iterative fitting based on the model behavior during the growth and micromanipulation simulations. The obtained value of k_{cort} is in a similar size scale that has been observed for other cells [13, 14, 15]. The junction strength (k_{junc}) was estimated to give balance against the cortical stiffness based on the cell shape as well as to prevent cells from separating too much during the micromanipulation simulations.

We used different values for the membrane elasticity (k_{mem}) depending on the simulation type. For micromanipulation, a larger value was used compared to the growth and optogenetic. This was for two reasons: 1) the short time scale was assumed to prevent fast remodeling of the membrane, and 2) a larger value was needed to keep the membrane vertices from accumulating to the following edge of the micromanipulated cell and thus forming long sections of the membrane without vertices that would have made the model unstable. The smaller value used for the rest of the simulation cases enabled the cells to dynamically change their shape and neighborhood.

The value for the contact repulsion strength (k_{cont}) was taken as the smallest repulsion value that would mostly prevent cell overlap in the simulations. The edge cell force constant (k_{edge}) was estimated by simulating micromanipulation without the substrate with epithelia of different sizes. We selected the value of k_{edge} that gave the same deformation for a smaller patch of epithelium compared to the same area in a larger patch of epithelium.

The value for the maximum membrane angle ($\theta_{mem,max}$) was chosen to give the cell enough freedom to form different shapes while preventing it from intersecting itself or forming narrow regions that could be form artifacts in the model. The maximum junction angle constant ($C_{junc,max}$) enabled the formation of junctions situations while also removing them to enable cells to slide against one another if needed. The focal adhesion breaking force ($F_{fa,max}$) was based on the observed movement after which the cells detached from the stiffer matrices in our experiments during micromanipulation and finding the focal adhesion force at the corresponding amount of micromanipulation movement in our simulations. The parameter needed for the strain-based remodeling of the cell perimeter, the perimeter remodeling rate (r_{per}) was obtained from Staddon et al. [6]

Table 1: The cell parameters values. G, growth; M, micromanipulation; O, optogenetics

Parameter name	Symbol	Value G	M	O	Unit
Normal membrane length	l_{mem}	1	1	1	μm
Junction rest length	l_{junc}	1	1	1	μm
Cell area elastic modulus	k_{area}	0.04	0.04	0.04	kg s^{-2}
Cortical tension	k_{cort}	1×10^{-3}	1×10^{-3}	1×10^{-3}	kg s^{-2}
Junction strength	k_{junc}	0.04	0.04	0.04	kg s^{-2}
Contact repulsion strength	k_{cont}	0.02	0.02	0.02	kg s^{-2}
Membrane elasticity	k_{mem}	1×10^{-4}	5×10^{-3}	1×10^{-4}	kg s^{-2}
Edge cell force constant	k_{edge}	–	5×10^{-3}	5×10^{-3}	kg s^{-2} μm^{-1}
Perimeter-tension constant	C_per	120	120	120	–
Maximum membrane angle	$C_{junc,max}$	60°	–	60°	–
Maximum junction angle constant	C_per	0.6	–	0.6	–
Focal adhesion breaking force	$F_{fa,max}$	10	–	10	nN μm^{-1}
Perimeter remodeling rate	r_{per}	0.00315	–	0.00315	s^{-1}

The growth simulations required multiple additional parameters related to the cell division and growth rate, and the used parameters are shown in Table 2. The division force constant (k_{div}) was estimated to give a good duration of cytokinesis. The parameter values related to the duration of different phases of the cell cycle – the division time (t_{div}), the maximum growth time ($t_{max,growth}$), and the maximum cytokinesis time ($t_{max,div}$) – were based on a combination of the biologically realistic time scales and giving cells time to relax stresses between divisions. The minimum cell area (A_{min}) was chosen to restrict the formation of small cells while still enabling the large size differences between cells. The values of constants related to the growth and division – the cell growth rate constant (C_{growth}), the cell growth force constant (C_{div}), the new cell area constant (C_{area}), and the division distance constant (C_{div}) – were chosen to obtain somewhat realistic and computationally stable cell growth rate and division. The values giving the size-dependent division probability of the internal cells, i.e. the base division rate ($\rho_{div,0}$) and the division rate exponent (α_{div}), were based on the literature [7]. The literature value of $\rho_{div,0}$ was found to produce too high division rate in our simulations, and thus we decreased its value tenfold to produce a better fit with our simulations and our cell size data.

The value of focal adhesion strength per membrane length (k_{fa}) was assumed to depend on the stiffness of

Table 2: The growth parameter values

Parameter name	Symbol	Value	Unit
Division force constant	k_{div}	1×10^{-8}	N
Division time (mean ± SD)	t_{div}	20 ± 4	h
Maximum growth time	$t_{max,growth}$	8	h
Maximum cytokinesis time	$t_{max,div}$	1	h
Division distance constant	C_{div}	0.5	–
New cell area constant	C_{area}	1.055	–
Minimum cell area	A_{min}	50	μm^2
Cell growth rate constant	C_{growth}	2	–
Cell growth force constant	C_{div}	0.2	–
Base division rate	$\rho_{div,0}$	5.67×10^{-13}	μm^{-6} s^{-1}
Division rate exponent	α_{div}	2.7	–

the underlying substrate. The values were obtained by fitting the model to the experimental cell and substrate deformation data from our micromanipulation measurements. With chosen mechanical parameters for the cells and the substrate, the value of k_{fa} was varied in the simulations and the computational deformation results were compared to the experimental data. The obtained values of k_{fa} for the different substrate stiffnesses from this fitting process are shown in Table 3. These values were defined per length of the membrane one vertex point describes and thus they are multiplied by l_{mem} to obtain the value used in the simulations. If the substrate stiffness was between the experimental stiffness values, the value of k_{fa} was linearly interpolated and if the stiffness was outside the experimental values, we assumed k_{fa} to remain constant with the closest fitted value.

Table 3: The values of the focal adhesions strengths for each substrate stiffness

Substrate stiffness (kPa)	Focal adhesions strength (kg s^{-2} μm^{-1})
1.1	5×10^{-4}
4.5	8×10^{-4}
11	1×10^{-3}

7.2 Substrate parameters

The values used for the substrate parameters in our simulations are described in Table 4. The rest length for the springs between the substrate points (l_{sub}) was chosen to reduce the simulation time as much as possible, while still having as high a resolution of the substrate as possible.

The values of Young's modulus constant (C_E) and the restorative force constant (C_{rest}) were estimated by using a finite element model created using Comsol Multiphysics (v. 5.4. www.comsol.com. COMSOL AB, Stockholm, Sweden). Comsol's solid mechanics module with the hyperelastic material model was used with a 2D geometry of a vertical cross-section of a block of polyacrylamide gel with a width of 200 μm and a height of 100 μm. The top surface corresponds to the horizontal plane in our substrate model, while the bottom surface was fixed to describe the attachment to the glass. A horizontal pulling force was applied to one of the vertices of the top of the block to simulate the gel block being pulled along this edge. We ran the corresponding simulations with the substrate model with the size of 200 μm by 200 μm and with a corresponding pulling force per length applied to one of the material edges. The substrate model was then fitted to the finite element model results by iteratively changing the values of C_E and C_{rest} with different Young's moduli to obtain their values.

The values for the honeycomb constants (α, β, and γ) were based on the desired Poisson's ratio for the substrate, which is close to 0.5 for polyacrylamide gels [12, 16, 17]. The constant for the repulsion length (C_{rep}) was chosen to minimize the effect of repulsion on the mechanical properties of the substrate under small deformations, while still preventing substrate collapse under large ones. The value for the constant for the edge points (C_{edge}) was fitting by simulating direct micromanipulation of the substrate without cells and comparing the deformation of a square substrate with the side length of 200 μm to that of a larger square substrate with the side length of 500 μm.

7.3 Simulation parameters

The parameters related to the simulation setup are presented in Table 5 for the different simulation types. The damping coefficients (η) were chosen to best describe the time scale of the events in each simulation type.

Table 4: The substrate parameter values

Parameter name	Symbol	Value	Unit
Substrate rest length	l_{sub}	2	μm
Young's modulus constant	C_E	7.62×10^{-6}	kg s^{-2} Pa^{-1}
Honeycomb constants	$[\alpha, \beta, \gamma]$	$[1, 1, 4]$	–
Restorative force constant	C_{rest}	2.9×10^{-4}	μm^{-2}
Repulsion length constant	C_{rep}	0.5	–
Edge point restorative constant	C_{edge}	100	–

The maximum time step (Δt_{max}) for each simulation type was selected to make the simulations stable. The scaling time ($\mathbb{T}$) and length ($\mathbb{L}$) used, together with η, to nondimensionalize the variables and parameters were selected based on the size scale of the cells and the time scale of the simulations with the substrate. The parameters related to the modification of the time step based on the maximum vertex ($d_{c,max}$ and $d_{c,low}$) and point movements ($d_{s,max}$ and $d_{m,low}$) were chosen based on the stability of the model during simulations. The junction remodeling time step (Δt_{junc}) was set higher for the growth simulations compared to the other simulation types to reduce the simulation time.

The model parameters related to the specific simulation types were obtained by fitting the model to our micromanipulation data (k_{mm}) and to optogenetic myosin activation data from the literature [6] ($C_{act,max}$). The latter was fitted to the single 20-min optogenetic activation data of the relative length of the junction sections (Fig. 2b in ref. [6]). The fitting was done by using a substrate stiffness of 0.5 kPa [18], corresponding to the approximate stiffness of the 2 mg mL^{-1} collagen gel used in the experiments [6].

Table 5: The cell parameters values. G, growth; M, micromanipulation; O, optogenetics

		Value			
Parameter name	Symbol	G	M	O	Unit
Damping coefficient	η	5	1×10^{-5}	0.5	kg s^{-1}
Maximum time step	$\Delta t_{c,max}$	60	1×10^{-4}	1	s
Scaling time	$\mathbb{T}$	1	1	1	s
Scaling length	$\mathbb{L}$	20	20	20	μm
Maximum cell movement	$d_{c,max}$	2	2	2	μm
Low cell movement	$d_{c,low}$	0.2	0.2	0.2	μm
Maximum substrate movement	$d_{s,max}$	–	0.5	0.5	nm
Low substrate movement	$d_{s,low}$	–	0.05	0.05	nm
Junction remodeling time step	Δt_{junc}	10	–	1	s
Micromanipulation force constant	k_{mm}	–	0.022	–	kg s^{-2}
Full optogenetic activation constant	$C_{act,max}$	–	–	4.5	–

7.4 Cell areas

The cell areas in our simulations were based on the segmented cell areas in our MDCK measurements. The area data were binned into 10-μm^2-wide bins and used to form a probability density function for the areas. The binned data were fitted with second-order Gaussian function using Matlab, and the resulting equation was

$$\begin{aligned} f(A) =&0.02902 \exp\left(-\frac{(A-103.3)^2}{164.4}\right) + 0.05107 \exp\left(-\frac{(A-79.61)^2}{369.0}\right) \\ &+ 0.07028 \exp\left(-\frac{(A-107.9)^2}{2101}\right) + 0.01260 \exp\left(-\frac{(A-164.0)^2}{6276}\right) \end{aligned}. \tag{28}$$

The histogram of the cell area data, as well as the probability density function obtained with Matlab curve fitting tool, are shown in Fig 4. The median value of the cell was used for the initial cell in the growth simulations was 105.4 μm^2.

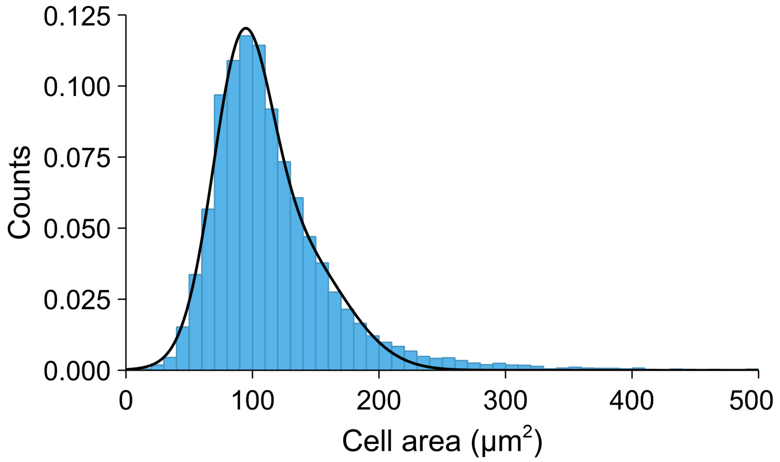

Figure 4: The MDCK II cell area distribution obtained from our experimental data binned into 10-μm^2-wide bins. The probability density function defined based on the distribution is shown by the black line and the equation shown in Eq. 28.

References

[1] Tervonen A. Epimech, v1.0.4. Zenodo. 2022;doi:10.5281/zenodo.6021250.

[2] Barton DL, Henkes S, Weijer CJ, Sknepnek R. Active Vertex Model for cell-resolution description of epithelial tissue mechanics. PLoS Computational Biology. 2017;13(6):1–34. doi:10.1371/journal.pcbi.1005569.

[3] Fletcher AG, Osborne JM, Maini PK, Gavaghan DJ. Implementing vertex dynamics models of cell populations in biology within a consistent computational framework. Progress in Biophysics and Molecular Biology. 2013;113(2):299–326. doi:10.1016/j.pbiomolbio.2013.09.003.

[4] Fletcher AG, Osterfield M, Baker RE, Shvartsman SY. Vertex Models of Epithelial Morphogenesis. Biophysical Journal. 2014;106(11):2291–2304. doi:10.1016/j.bpj.2013.11.4498.

[5] Cooper L, Maddock S. Preventing Collapse Within Mass-Spring-Damper Models of Deformable Objects. In: The 5th Int. Conf. in Central Europe on Comput. Graphics and Vis. 1; 1997. p. 196–204.

[6] Staddon MF, Cavanaugh KE, Munro EM, Gardel ML, Banerjee S. Mechanosensitive Junction Remodeling Promotes Robust Epithelial Morphogenesis. Biophysical Journal. 2019;117(9):1739–1750. doi:10.1016/j.bpj.2019.09.027.

[7] Puliafito A, Primo L, Celani A. Cell-size distribution in epithelial tissue formation and homeostasis. Journal of the Royal Society, Interface. 2017;14(128):1–9. doi:10.1098/rsif.2017.0032.

[8] Hertwig O. Das problem der Befruchtung and der Isotropie des Eies, eine Theorie der Vererbung. Jena: Fischer; 1884.

[9] Tamulonis C, Postma M, Marlow HQ, Magie CR, de Jong J, Kaandorp J. A cell-based model of Nematostella vectensis gastrulation including bottle cell formation, invagination and zippering. Developmental Biology. 2011;351(1):217–228. doi:10.1016/j.ydbio.2010.10.017.

[10] Ostoja-Starzewski M. Lattice models in micromechanics. Applied Mechanics Reviews. 2002;55(1):35–59. doi:10.1115/1.1432990.

[11] Day AR, Snyder KA, Garboczi EJ, Thorpe MF. The elastic moduli of a sheet containing circular holes. Journal of the Mechanics and Physics of Solids. 1992;40(5):1031–1051. doi:10.1016/0022-5096(92)90061-6.

[12] Snyder KA, Garboczi EJ, Day AR. The elastic moduli of simple two-dimensional isotropic composites: Computer simulation and effective medium theory. Journal of Applied Physics. 1992;72(12):5948–5955. doi:10.1063/1.351903.

[13] Winklbauer R. Cell adhesion strength from cortical tension - An integration of concepts. Journal of Cell Science. 2015;128(20):3687–3693. doi:10.1242/jcs.174623.

[14] Foty RA, Steinberg MS. The differential adhesion hypothesis: A direct evaluation. Developmental Biology. 2005;278(1):255–263. doi:10.1016/j.ydbio.2004.11.012.

[15] Pasternak C, Spudich JA, Elson EL. Capping of surface receptors and concomitant cortical tension are generated by conventional myosin. Nature. 1989;341(6242):549–551. doi:10.1038/341549a0.

[16] Boudou T, Ohayon J, Picart C, Tracqui P. An extended relationship for the characterization of Young's modulus and Poisson's ratio of tunable polyacrylamide gels. Biorheology. 2006;43(6):721–728.

[17] Takigawa T, Morino Y, Urayama K, Masuda T. Poisson's Ratio of Polyacrylamide (PAAm) Gels. Polymer Gels and Networks. 1996;4(1):1–5. doi:10.1016/0966-7822(95)00013-5.

[18] Joshi J, Mahajan G, Kothapalli CR. Three-dimensional collagenous niche and azacytidine selectively promote time-dependent cardiomyogenesis from human bone marrow-derived MSC spheroids. Biotechnology and Bioengineering. 2018;115(8):2013–2026. doi:10.1002/bit.26714.
